# A Review of the Host Plant Location and Recognition Mechanisms of Asian Longhorn Beetle

**DOI:** 10.3390/insects14030292

**Published:** 2023-03-17

**Authors:** Fei Lyu, Xiaoxia Hai, Zhigang Wang

**Affiliations:** Key Laboratories for Germplasm Resources of Forest Trees and Forest Protection of Hebei Province, College of Forestry, Hebei Agricultural University, Baoding 071001, China

**Keywords:** *Anoplophora glabripennis*, host plant list, host kairomone, OBP, microbial symbionts, visual ecology

## Abstract

**Simple Summary:**

The Asian longhorned beetle (ALB), *Anoplophora glabripennis* Motschulsky, is a destructive pest in its native habitat and one of the most serious invasive alien species in North America and Europe, causing substantial economic and ecological losses. In order to explore effective monitoring and management strategies, we summarize and create a comprehensive list of host plants, including 209 species (cultivars) that have been damaged by ALBs. Thus far, 143 olfactory protein genes have been found in ALBs. Host kairomones were preferentially bound to ALB recombinant odorant-binding proteins (OBPs), but the function of most OBPs is still unclear. Microbial communities may help ALBs degrade host plants. We analyzed the trapping effect of combined host kairomones and sex pheromones and found that trapping numbers are limited in the field. Therefore, we discussed host plant location behavioral processes from new perspectives and found that multiple cues are used to locate and recognize host plants. Overall, we suggest that further research should contribute to understanding the host resistance mechanism, microbial community influence mechanism, and visual cue recognition mechanism of host plants. This research may provide effective monitoring and management strategies for ALBs.

**Abstract:**

The Asian longhorn beetle (ALB), *Anoplophora glabripennis* Motschulsky, is a polyphagous xylophage with dozens of reported host tree species. However, the mechanisms by which individuals locate and recognize host plants are still unknown. We summarize the current knowledge of the host plant list, host kairomones, odorant-binding proteins (OBPs) and microbial symbionts of this beetle and their practical applications, and finally discuss the host localization and recognition mechanisms. A total of 209 species (or cultivars) were reported as ALB host plants, including 101 species of higher sensitivity; host kairomones were preferentially bound to ALB recombinant OBPs, including *cis*-3-hexen-1-ol, *δ*-3-carene, nonanal, linalool, and *β*-caryophyllene. In addition, microbial symbionts may help ALB degrade their host. Complementarity of tree species with different levels of resistance may reduce damage, but trapping effectiveness for adults was limited using a combination of host kairomones and sex pheromones in the field. Therefore, we discuss host location behavior from a new perspective and show that multiple cues are used by ALB to locate and recognize host plants. Further research into host resistance mechanisms and visual signal recognition, and the interaction of sex pheromone synthesis, symbiont microbiota, and host plants may help reveal the host recognition mechanisms of ALBs.

## 1. Introduction

Urban landscape and ornamental tree species provide multiple services for city residents, including recreational and tourism opportunities for humans and habitats for diverse biotic communities, promoting biodiversity, climate regulation, and even timber and nontimber production [1]. Insects are the most common organisms in forests, and they play an important role in maintaining ecosystem balance [2]. In contrast, some insects attack and kill healthy trees, disturbing the dynamic balance of the ecosystem and reducing beneficial ecological services. In particular, nonnative insects can threaten biodiversity and affect the ecological function of native organisms.

The Asian longhorned beetle (ALB), *Anoplophora glabripennis* Motschulsky, was initially found in private gardens, public parks, and street trees, and is one of the most important nonnative invasive species in Europe and North America. ALB can attack healthy trees, causing tree mortality and substantial economic and ecological losses. In a widespread ALB outbreak, the potential economic loss could exceed USD 1 trillion, and approximately 35% of urban trees could be damaged across the USA [3]. The gross potential impact from ALB introduction was estimated to have exceeded CDN 12 billion annually in eastern Canada, affecting roadside greenways, standing timber and maple food product values [4]. ALBs are polyphagous xylophages native to Asia, and dozens of deciduous tree species have been reported as host plants of the ALB [5,6]. Several of its host trees are important sources of industrial materials in North America; for example, *Acer saccharum* Marshall, which is a source of maple syrup and maple sugar. Moreover, the overwhelming majority of tree species are ornamental and are used to decorate the living spaces of houses, streets, gardens or other places to provide a comfortable environment for residents. Other hardwood lumber species are used to make furniture and flooring. 

Many management strategies have been widely used to control this damaging pest. Potential biological control agents for ALB include entomopathogenic fungi and bacteria, parasitic nematodes, parasitoids, and predators [7]. The entomopathogenic fungi *Beauveria brongniartii* Petch and *Metarhizium brunneum* Petch have a high mortality rate for ALB, but the virulence of fungi is limited by lower ambient temperatures [8,9]. More than 35 species of ALB-associated parasitoids, including more than 20 in China and Korea, 8 ectoparasitoids in Europe, and 7 groups of branconid parasitoids in North America [3], have been reported in the native and nonnative ranges of this insect [7,8,10,11]. These parasitoids not only attack ALB and *Anoplophora chinensis* (Citrus longhorn beetle, CLB), but also parasitize other woodboring pests. Therefore, their mass release has not been considered to date [3]. For example, *Dastarcus helophoroides* is an important natural enemy of ALB in China, but it attacks other species of longhorn beetles, including *Monochamus alternatus* (Hope), *Massicus raddei* Blessig, and *Batocera horsfieldi* (Hope) [10,11]. Trunk or soil injections and sprays with pesticides have been applied frequently in China, the United States, and Europe [5]. However, greater utilization of pesticides reduces the regional biodiversity of insects in freshwater and terrestrial systems, and results in biomagnification of toxic substances within the food web, potentially affecting human and animal health [12] and contributing to global insect pollinator declines [13]. Eradication plans for ALBs have been successful, but this approach is also expensive. Many countries have considered using containment instead of eradication for ALB [3]. For example, the estimated cost of eradication programs was approximately USD 537 million between 1996 and 2013 in the USA [3]. Therefore, there is an urgent need for further studies on more environmentally friendly control strategies to manage this alien pest [14].

The location and selection of host plants is critical in the life cycle of ALB and plays an important role in integrated pest management (IPM). Adult ALB requires nutritional supplements for sexual maturation and oviposition. Twigs and branch bark are the main food sources of ALB, and foliage and larger branches are rarely harmed by these beetles [5]. In addition, the location and recognition of oviposition hosts by adult female ALBs is also critical because the larvae are usually unable to move between hosts [15]. The nutrition of the host plant directly influences the survival, longevity, and fecundity of larvae and adults [16,17]. Suitable larval hosts were located by female beetles primarily through olfactory cue identification [3,18,19], and many host kairomones were identified from host and nonhost plants (see Section 2.2). We found that the visual cues of host plants also influenced host plant identification [20]. Host plant branch bark color signals significantly increase the responses of ALB adults to host plant odor [20]. However, semiochemical-based trap lures have not achieved operational efficacy in the detection and management of ALB to date (see Section 4.2). Therefore, the mechanism by which ALBs locate host plants requires further clarification from a new perspective. 

The aims of this work are (1) to create a comprehensive list of host plants, thus providing a theoretical basis for further research on the interaction of ALBs and host plants, (2) to summarize the relationships between host kairomone types and odorant-binding proteins (OBPs) and the interaction between host plants and microbial symbionts of ALBs, (3) to analyze the influence of ALB population density on mixed forests and the effects of host kairomones on the ability to trap this pest, and (4) to provide a new perspective on the behavior used by ALBs for locating host plants. This review will provide a valuable reference and context for understanding the interactions between this beetle and its host plants, as well as new strategies for ALB monitoring and management.

## 2. Host Plant List and Host Kairomones

### 2.1. Host Plant Lists

We found that at least 209 species (or cultivars) from 41 genera, 21 families, and 10 orders have been reported as host plants of ALBs in Asia, North America, and Europe [6,17,18,21,22,23,24,25,26,27,28,29,30,31,32,33,34,35,36,37,38,39,40,41,42,43,44,45,46,47,48,49,50,51,52,53] (Tables S1 and S2). ALBs infest broad-leaved tree species mainly in the genera *Populus*, *Acer*, *Salix*, *Ulmus*, *Aesculus*, and *Betula*; approximately 75.12% of the host plant species belong to these six genera, including 41.15% of *Populus* species, 12.92% of *Acer* species, 9.56% of *Salix* species, 5.74% of *Ulmus* species, 2.87% of *Aesculus* species, and 2.87% of *Betula* species (Table S2). In particular, approximately 86 species or cultivars that belong to *Populus* are susceptible to damage, and 95.35% (82/86) of these *Populus* species have been recorded in China (Table S2). In China, *Populus* and *Salix* are the most commonly infested genera, while *Acer* is the most commonly infested tree genus in North America and Europe [5,6]. A total of 162, 36, and 26 tree species have been reported as hosts in China, North America, and Europe, respectively. Based on information about the development of adult ALBs on these plants, reported in the literature from 1992 to 2022, which is available in the Web of Science and China National Knowledge Infrastructure (CNKI), these tree species were classified into four types:

I: High-sensitivity (HS) plant species are those on which the ALB has been reported to complete its life cycle (from oviposition to the emergence of new beetles); these plants were recorded as highly sensitive or very good host plants [21,23,34]. It has been reported that ALBs are able to complete their life cycle on 171 species of plants, including 101 species of highly sensitive host plants, and 70 species of moderate-sensitivity plants (Tables S1 and S2).

II: Moderate-sensitivity (MS) plant species are those on which the ALB completes its life cycle but that have not been recorded as highly sensitive or very good host plants.

III: Partial-sensitivity (PS) plant species are those on which the ALB completes part of its life cycle, including feeding and oviposition, but for which exit holes have not been recorded. Tree species classified as PS and S are rarely reported, with 11 and 27 tree species, respectively.

IV: Low-sensitivity (LS) plant species are those on which only feeding or oviposition of the ALB has been recorded (without exit holes) [26].

The results showed that 101, 70, 11, and 27 tree species belonged to these four categories, respectively. The host plant species differ significantly by region; for example, 78, 63, 5, and 17 species belonged to the HS, MS, PS and LS categories in China, respectively; 16, 9, 7, and 4 species were classified into the HS, MS, PS, and LS categories in North America, respectively; and 17, 4, 1, and 4 species were classified into the HS, MS, PS, and LS categories in Europe, respectively (Table S2).

### 2.2. Host Plant Kairomones

Phytophagous insects locate and recognize host plants via multiple cues, such as odor (olfactory cues), color, size and shape (visual cues), and even auditory and gustatory signs [54,55,56,57,58,59,60,61,62,63]. Many previous studies focused on olfactory cues in adult ALBs, and large quantities of volatile organic compounds have been identified from host and nonhost plants (Table 1). Approximately 13 attractive and 9 repellent chemical compounds were found for both male and female adults. The attractive chemical compounds include four alcohols, one aldehyde, seven olefins, and one ester, and the repellants include two alcohols, five olefins, one ketone, and one ester (Table 1). These substances have the following characteristics.

(1) The effects of single and mixed green leaf volatiles were significantly different in their abilities to attract ALBs; for example, *cis*-3-hexen-1-ol was significantly more attractive than hexenal, trans-2-hexen-1-ol and hexanoic acid, but a mixture of 1-pentanol, 1-butanol, *cis*-3-hexen-1-ol, 2-pentanol and 2-butoxy-ethanol was preferred over single compounds by adults [66].

(2) Different concentrations of chemical compounds have different trapping effects in adults; low concentrations (0.0004–0.004 mol/L) may not be attractive to adults, while high concentrations (0.04–2 mol/L) may be attractive; this has been observed for R-*α*-pinene, S-*α*-pinene, S-*β*-pinene, phellandrene, and other compounds [44]. Moreover, a single chemical compound may be an attractant for adults when combined with host odors, whereas when combined with nonhost volatiles, it may be a deterrent, such as *β*-caryophyllene [67].

(3) The same chemical compounds have different effects on female and male adults; for example, myrcene and 3-carene are not attractive to females at concentrations of 0.0004–2 mol/L but have a positive trapping effect on males when used at concentrations of 0.4 mol/L and 2 mol/L [44].

## 3. Interaction of ALBs, Microbes and Host Plants

### 3.1. Odorant-Binding Proteins of ALB to Recognize Host Plants

OBPs play a vital role in the communication between insects and odorant molecules from host plants, conspecifics and environments and are widely used to perceive and carry odorant molecules to odorant receptors in the hydrophilic sensillum lymph [71]. A total of 61 OBP genes have been identified and classified into four types, including 10 *Agla*OBPs classified as classical OBPs; 29 *Agla*OBPs lacking C2 and C5, classified as minus-C OBPs; 15 *Agla*OBPs belonging to Antennae-binding proteins subfamily (ABP II); and 1 *Agla*OBPs classified as plus-C OBPs based on genome projects and transcriptomic data of ALBs [72]. The number of OBPs is greater than that reported for other species of Coleoptera, including the Cerambycidae *M. alternatus* Hope, *Saperda populnea*, *M. saltuarius*, and the other beetles, *D. helophoroides*, *Dendroctonus ponderosae*, and *Ips typographus* [73,74,75,76]. ALB is a polyphagous species, and its hosts comprise 209 broad-leaved plants (cultivars) (Table S2). Plants differ in their chemical compounds, and diversified OBPs may contribute to the recognition of various food types using complex chemosensory systems. Twelve *Agla*OBP genes are expressed specifically in the antennae, including *Agla*OBP3, *Agla*OBP4, *Agla*OBP18, *Agla*OBP21, *Agla*OBP33, *Agla*OBP41, *Agla*OBP45, *Agla*OBP46, *Agla*OBP47, *Agla*OBP48, *Agla*OBP50, and *Agla*OBP53; in particular, *Agla*OBP3, *Agla*OBP18, *Agla*OBP21, *Agla*OBP33, *Agla*OBP41, *Agla*OBP45, and *Agla*OBP47 are highly expressed in male antennae, and may be used to recognize sex pheromones [72].

The fluorescent competitive binding assay plays an important role in detecting the binding efficacy of OBPs. This can show the conjunction ability of OBPs with the molecules of host kairomones. There is a significant difference in the conjunction ability of different OBPs with the same chemical molecule. Although a total of 61 OBP genes have been found in ALBs, research on the effective binding of OBPs involved only *Agla*OBP1, *Agla*OBP12, *Agla*OBP45, and *Agla*OBP46 (Table 2 and Table S3) [77,78,79]. The results showed that multiple OBPs may be involved in the host location and recognition process. Moreover, the abovementioned four recombinant *Agla*OBPs show obvious preferential binding to volatiles from host plants of ALBs; for instance, *cis*-3-hexen-1-ol, *δ*-3-carene, nonanal, linalool, and *β*-caryophyllene have been found in the host plants sugar maple, striped maple, and horse chestnut [67].

The trapping effects on ALB of some semiochemical constituents that have higher binding affinities with *Agla*OBPs, for example, heptanal, and butyl caproate, have not been tested in the laboratory or field. These substances may improve the current situation in which the effect of trap lures is limited to adult ALBs (see Section 4.2) [79]. Therefore, the binding effectiveness of the other *Agla*OBPs with semiochemicals should be tested using a fluorescent competitive binding assay, and the experimental results may provide a new perspective for the integrated management of ALBs.

### 3.2. Collaboration with Microbes to Degrade Host Plant Tissue

Beneficial gut microbes enhance the fitness of most living organisms, particularly wood-feeding insects [82]. ALBs typically lay eggs along the upper trunk and main branches of a tree. Females usually chew a distinct funnel-shaped and T-shaped oviposition pit through the bark at the phloem–cambium interface, and then inject a single egg into the bark [5]. Larvae create a feeding gallery and oval-shaped tunnel in the phloem and xylem, and all the larval nutrition is derived from the phloem and xylem of the host plant. Glucose is a predominant wood sugar, but it is reserved on the complex polysaccharides in the phloem and xylem, including cellulose, hemicellulose, callose, and pectin, which are suboptimal substrates that are nutritionally deficient and inherently difficult to digest. Nitrogen, fatty acids, sterols, and vitamins are also extremely limited in woody tissues [83,84]. Therefore, it is extremely challenging to acquire sufficient nutrients to complete development when digesting these substances [85]. The midgut transcriptome indicated that ALBs can produce several enzymes associated with cell wall digestion, detoxification, and nutrient extraction, but few transcripts were identified and predicted to encode enzymes of lignin degradation or synthesis of essential nutrients, indicating that other enzymes may be provided by microorganisms in the gut to enable the survival of these larvae in woody tissue [85]. 

In addition, plant secondary metabolites, such as diterpenic acid, flavonoids, phenols, and alkaloids play an important role in insect resistance [51,86] and the contents and assemblages of these substances vary with plant species. In addition to enzymes produced by the digestive tract, beneficial microbes in the insect gut are associated with the degradation of plant insect-resistant substances [87]. A previous study showed that the contents of flavonoids, simple phenols, coumarin and its derivatives were higher in the xylem of *Fraxinus chinensis* than in that of *F. pennsylvanica* [51]. Moreover, when ALB fed on *F. chinensis*, the intestinal bacterial community of ALB involved in the metabolism of these substances, including *Enterococcus* and *Raoultella*, was significantly larger [51]. This suggests that these microbial symbionts in the gut of insects produce a number of enzymes to degrade these toxic substances, thereby improving fitness and expanding the host’s ecological niche [88]. ALBs attack and kill healthy trees and have a wide range of host tree species (Table S2) [5]. Sophisticated abilities should have evolved in ALBs to evade host plant defenses and they should possess extensive suites of enzymes involved in digestive proteinase inhibition, detoxification of plant metabolites, and disruption of jasmonic acid signaling pathways [89]. Therefore, to understand the expansion of the ecological niche of ALBs, the important role of microbial symbionts in these metabolic processes and how they lead to the evolutionary success of ALBs with a variety of host plants need to be further researched.

## 4. Practical Applications of Host Plants and Host Kairomones

### 4.1. Mixed Forest

Push–pull strategies, in which the behaviors of insect pests and their natural enemies are manipulated using special stimuli, are a useful tool for IPM programs aiming to reduce the use of pesticides [60]. One means of manipulation is to repel pests away from protected crop plants by using plants that are unattractive or unsuitable for the pests. The host plant list of insect pests is a key factor in this strategy. Therefore, an explicit host plant species list provides a strategy for managing the ALB population. In 1999, an ecological management model for the cooperative planting of tree species with different resistance levels was used to manage the population density of ALBs in China [90]. This management method had two main components, including removing pest-infected tree trunk sections and grafting pest-resistant tree species onto the residual root of the infected tree species. Thus, a protection zone was built using ALB-resistant and ALB-tolerant tree species to prevent the diffusion of ALBs, and the percentage of damaged trees was reduced from 98.7% in 1976 to 3.8% in 1999. As a result, the output value of the woodland increased sevenfold.

Bottom-up effects could be mediated by first-trophic-level variables, impacting the survival, development, behavior, and population dynamics of insect pests and crop yield. The use of first-trophic-level variables—that is, a proper configuration of resistant and sensitive plant species—triggered changes in crop diversity, insect pest habitat, fertilization, volatile compounds and other factors, notably influencing pest populations and potentially enhancing pest control [61]. A clarified list of host plants can also be used to select appropriate tree species in mixed forests to decrease the damage caused by ALBs. An investigation showed that the ALB-induced damage rate decreased with an increase in ALB-resistant tree species in a mixed forest, probably because the phenological phase changes in ALB-resistant tree species relative to those of ALB-sensitive trees complicate the background of chemical cues [91]. Moreover, adult ALB mating frequency and landing frequency on host plants decreased in mixed forests composed of *Ailanthus altissima* and *P. bolleana* [92].

### 4.2. Trapping Technique

Insect pheromones and plant volatile compounds are known to be key factors in species communities. Plant semiochemicals can prompt insects to exhibit a wide range of behavioral responses. For example, host plant volatiles can enhance insects’ responses to sex pheromones [93,94]. Studies have shown that adult ALBs are more attracted by a combination of host kairomones and sex pheromones than by single host volatiles or pheromones [94]. This characteristic has been used by pest management experts to improve monitoring techniques and pest control strategies. Host kairomones, which mainly contain *cis*-3-hexen-1-ol, *β*-caryophyllene, linalool, *delta*-3-carene and camphene, and sex pheromones, which mainly include volatile pheromones from male- (4-(n-heptyloxy)butanal-1-ol, 4-(n-heptyloxy)butanal) and female-produced long-range pheromones (heptanal, nonanal, and hexadecanal), have been used to monitor populations in the early stages of invasion [95,96] (Table 3).

Although the combination of host volatiles and sex pheromones has been found to attract more ALB adults than the control, the number of traps applied in the field is still limited (Table 3). The response ratio of adults was approximately 40–90% of the total mean trap catches per week in traps including sex pheromones and/or host kairomones identified using a Y-tube olfactometer in the laboratory; moreover, the mean trap catches per week was approximately 40% of total test insect samples in four traps in a greenhouse [68]. Relative to that in the laboratory and greenhouse, the trapping effect was significantly decreased when male- and female-produced pheromones and/or volatile compounds from host plants were used in the field (Table 3). For example, 42 beetles were trapped by 90 flight intercept panel traps from 23 July to 19 August in Harbin, China [99]. In addition, the recapture rate of adults was 5.14% of the total number of released adults according to the “mark-release-recapture” technique when male-produced pheromones in the 200 m range were used in the field in Hengshui, Hebei Province, China [42]. However, approximately 120 beetles were caught every 3 h by three researchers over a one-week period in high-density populations in Baoding, Shijiazhuang, Cangzhou, and Hengshui, Hebei Province, China. The manual capture of ALBs in the field corresponds with the population size derived from the “mark-release-recapture” technique. Therefore, a highly attractive trapping device for ALBs with an effective trapping lure, shape and color is urgently needed.

In previous studies, limited trapping may have been affected by many factors, such as the test site, the shape and color of the trap, and the composition of the lure. 

(1) Due to experimental site selection, the mean number of ALB adults caught may be very low in the same traps. The population density of ALB larvae and pupae varied by location and tree species [103]; for example, the density in Jilin Province was higher than that in Gansu, Shaanxi, Hebei and Beijing [14]. We also found that adult population size exhibited a significantly skewed distribution in the field investigation. The number of captured individuals was higher in the high-density population than in the low-density population in Hebei, China. Therefore, the test site should also be considered in field experiments.

(2) There was a significant difference in the mean trap catch per lure between different types of traps, particularly among traps differing in shape and color. Intercept panel traps caught more adults per week than the other traps, including hand-made screen sleeve traps, Plum curculio traps and Lindgren funnel traps, in greenhouses when baited with male-produced sex pheromone blends and (*Z*)-3-hexen-1-ol, whereas screen sleeve traps were most attractive when baited with (-)-linalool [68]. In addition, the average number of beetles captured by brown traps was significantly higher than that captured by noncolor-modified traps when the content of 2-pentanol was increased, but the catch capacities still did not significantly increase [102].

(3) The composition of the lure is also a key factor in trapping ALBs. Although approximately 13 kinds of attractive host plant volatiles and 20 kinds of sex pheromones have been found in recent years [80,81,95,96,98,104,105] (Table 2 and Table S4), there is no sufficiently strong attractive chemical available for practical application in the field, similar to observations for other cerambycid species, especially early in the invasion process [105]. For example, long-range female-produced sex pheromones were more significantly attractive to adult ALBs than combinations of host kairomones and linalool oxide [98], but trapping capacities remained limited (Table 3). Therefore, it is necessary to further discuss the host plant location and recognition behavior of ALBs, and this process may be more complicated than previously speculated.

## 5. Host Plant Location and Recognition Behavior Hypothesis

**Figure 1:**
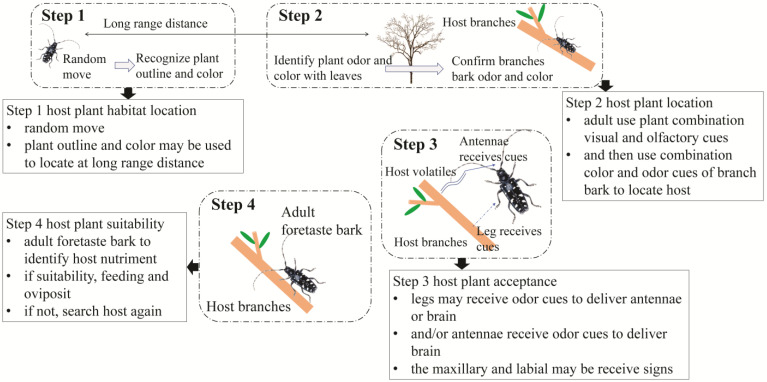
Hypothesized host plant location and recognition behavior process in ALB.

Although considerable achievements have been made in chemical ecology research, the host location and recognition mechanisms of ALBs are still unclear. During host location in parasitoids, the host search process is divided into four steps: host habitat location, host location, host acceptance, and host suitability [106]. We suggest that host plant location and recognition in ALBs are also stepwise processes, similar to the process followed by parasitoids. First, adult females and males must identify plant profiles and colors over long distances; then, they must distinguish leaf color and olfactory cues and identify branch bark color and odors at close range. Adults collect bark chemical cues with their legs and deliver bark chemicals from their legs to their antennae and/or taste the bark after they have landed on the tree. Finally, adults decide whether to feed or lay eggs on the plant based on their nutrition after the “try to taste” process (Figure 1). We suggest that host plant location and recognition in the ALB are incremental and that not only odor cues but multiple types, such as visual, olfactory, gustatory, and even tactile cues, are used.

(1) Host plant habitat location: First, adults randomly move or fly to a host plant over a long distance. Subsequently, the outline and color of the plant may be considered important recognition cues when the adult is relatively far away from the plant. In the field, we found that adults could rapidly travel to plants instead of into a lake when host plants were grown near the lake, and adults could fly directly over a road and land on a plant on the other side of the road when host plants were planted on both sides of the road. These observations confirmed that the outline and color of plants play an important role in host plant location over long distances. Li [107] also suggested that the greenness of plants plays an important role in host plant location over long distances. In fact, the influence of the environment on the dispersal of volatiles should be considered, and host recognition by insects via olfactory cues is disrupted by complex volatile backgrounds [108]. This phenomenon is often suggested as the reason why visual cues from host plants are more effective than olfactory cues over long distances. Therefore, visual cues may play a more important role than olfactory cues in the process of locating host plants.

(2) Host plant recognition: The host plant habitat, comprising a mixture of host and nonhost plants, is confirmed by adults, but how they differentiate optimal host plants for growth and development from nonhost plants has not yet been clarified. We suggest that ALB first uses a combination of visual and olfactory signals from branches with leaves to locate host plants within an 80 cm range [109]; then, ALB uses combined color and odor cues from branch bark to locate and recognize the host plant [20]. There was no significant difference in the first orientation of adults when comparing visual or olfactory cues from *A. negundo* and *Pinus bungeana* branches with leaves; however, there was a significant difference between host and nonhost plants when a combination of visual and olfactory cues was provided for adults in the 80 cm range [109]. This result suggested that the effective attractive range of combined visual and olfactory cues is greater than that of single visual or olfactory cues for adults.

(3) Host plant acceptance: We speculate that a combination of bark color and olfactory cues is used to probe and evaluate the fitness of host plants contacted by adults; afterward, the thoracic legs may receive host bark chemical cues and deliver bark chemicals to antennae and/or taste the bark via gustatory receptors in the palps that contact the substrate [20]. Moreover, we found that the start time of grooming between the antennae and pro- or mesothoracic tarsi lags behind that of the first visit to a host plant, so we suggest that the thoracic legs also receive and transfer chemical cues to antennae or the brain to recognize host plants based on the chemical material collected on the feet [110].

(4) Host plant suitability: We suggested that the physicochemical properties of the host bark and the composition of host secondary metabolites were the key factors determining whether adult ALBs would feed on selected plants after the “try to taste” process using the gustatory sensory system in the final step. In previous research, we found that individual male and female beetles fed on cut branches of *A. altissima*, and the feeding areas of this species were very small: approximately 0.03–0.04 cm^2^ per 12 h, on the branch bark of *A. negundo* and *A. altissima* without leaves. However, *A. altissima* is a resistant species according to the results of Cao et al. [111]. Therefore, we suggest that the “try to taste” process is also a crucial part of host plant recognition.

## 6. Further Research Directions on Interaction of ALBs and Host Plants

ALB is a high-risk species in its native and nonnative ranges and seriously endangers the economic and ecological value of forests in North America, Europe, and China [3,5]. ALB have been reported to be capable of attacking 209 species (or cultivars) from different families and exhibit exceptional host adaptability. Among the biochemical defenses, plant secondary metabolites are the most diverse and effective weapons to defend against pest and pathogen damage [112,113]. Phenolic glycosides and flavonoids are important plant secondary metabolites that suppress the foraging and oviposition of pests and strongly influence the growth, development, and behavior of insect herbivores [114,115,116]. However, generalist insects can overcome the effects of phenolic glycosides, even in particular insect species that use phenolic glycosides as feeding and oviposition stimulants [117], such as *Galerucella lineola*, *Lochmaea capreae*, *Megaselia opacicornis*, and *Nematus oligospilus* [118,119,120]. Furthermore, horizontal gene transfer (HGT) is widely recognized for prokaryotes and eukaryotes and can lead to the exploitation of new resources and niches [121,122,123,124,125,126]. The whitefly, *Bemisia tabaci* (Gennadius), is extremely polyphagous, with more than 600 reported host plant species [127]; the species has been shown to hijack a plant detoxification gene, BtPMaT1, which enables whiteflies to neutralize phenolic glucosides [114]. ALB is also extremely polyphagous; nevertheless, how this beetle neutralizes plant toxins and host resistance mechanisms remains unknown.

Trapping lures play a crucial role in surveys and management programs for insect pests [94]. To quickly detect the population dynamics of ALB and reduce damage, a high-efficiency trapping device and a sustainable management strategy for ALB are urgently needed. At present, semiochemical attractants, including mainly sex pheromones and host kairomones, are used to detect the population dynamics of Cerambycidae [128,129,130,131,132], but the attractant effects on some species are limited. In addition, traps combining female-produced aldehydes and host kairomones captured more ALBs than control traps, but the mean trap catches were only 0.7–7 per trap per week [98]. Therefore, some key sex pheromones require identification.

There is a great deal of research indicating that microbial symbionts can directly modulate their host’s biosynthesis of pheromones and other chemical components or provisioning of precursors, thus mediating mate choice decisions and some social behavior [133]. Some actions of bacterial manipulators of insect reproduction were observed, such as parthenogenesis, male killing, feminization and cytoplasmic incompatibility, to promote their own spread within a host population, including *Wolbachia*, *Spiroplasma*, *Cardinium*, *Rickettsia*, and some *Bacteroidetes* [134]. In the saw-toothed grain beetle *Oryzaephilus surinamensis*, the *Bacteroidetes* symbiont supports cuticle synthesis to influence its cuticular hydrocarbon profile, and hence may modulate the release of sex pheromones [133,135]. After feeding on *P. alba* var. *pyramidalis* and *S. babylonica*, *Wolbachia* was the predominant bacteria in the gut of larvae, while *Bacteroidetes*, *Firmicutes*, and *Actinobacteria* were predominant in larvae fed on a preferred host (*A*. *saccharum*) [136,137]. However, whether *Wolbachia* and *Bacteroidetes* influence the synthesis of cuticular hydrocarbons and mating of ALB has yet to be demonstrated. Further research focusing on the interaction of sex pheromone synthesis and symbiont microbiota may contribute to the development of trapping lures.

A total of 143 olfactory-related protein genes, including 61 OBPs, 12 chemosensory proteins (CSPs), 37 odorant receptors (ORs), 4 ionotropic receptors (IRs), 11 gustatory receptors (GRs), 1 odorant-degrading enzyme (ODE), 3 sensory neuron membrane proteins (SNMPs), and 14 pheromone-degrading enzymes (PDEs), were identified by antennal transcriptome analysis [79,138]. This is fewer than the 151 genes in *A. chinensis* and 166 genes in *Semanotus bifasciatus*, but greater than the number of chemosensory receptor genes in the other cerambycids [75,76,139,140]. However, the functions of these sensory proteins are still unclear. Fluorescence competitive binding experiments can reveal the specific molecules with which OBPs bind. The results of previous studies suggested that *Agla*OBP45 can significantly bind with contact and trail pheromones of females (Table 3), but the binding capacity with sex pheromones from males was not reported in previous studies. *Agla*OBP47, *Agla*OBP48, and *Agla*OBP53 were particularly highly expressed in female antennae, and these OBPs may play an important role in the recognition of host plant kairomones, male-produced sex pheromones, mates, and/or suitable oviposition sites by females [72]. Thus, some OBPs that bind to sex pheromones need to be further explored, and the results will likely provide an appropriate lure for trapping ALB in the field.

Color vision is widespread among insects, but species differ in their spectral sensitivities [54]. Cavaletto et al. [141,142] suggested that trap color is also a key visual cue that can strongly increase the attractiveness of baited traps to longhorn beetles. Monitoring programs should not rely exclusively on black traps, and other trap colors can likely strongly improve the chance of trapping native and exotic longhorn beetles [141]. Our study also showed that the combination of bark color and odor cues of branches was used to locate host plants, and forest green paperboard enhanced the attractiveness of host branch volatiles in the laboratory [20]. This result suggested that color is a potential influencing factor in trapping effectiveness. Moreover, flight intercept traps with chemical attractant baits and/or special wavelengths of light are used to survey target native and nonnative forest insects, improving the trapping effect [2,143]. However, there was no significant improvement in the trapping effect when using brown intercept panel traps baited with 1- or 2-pentanol, and the average capture frequency was 1.521 beetles per trap per week [102]. Therefore, whether the capture capacity of semiochemicals (sex pheromones and host kairomones) for ALB adults can be enhanced by green or even other colors needs to be further researched in the field.

Light traps play an important role in pest management and are used extensively in IPM [54,143]. Many pests, especially nocturnal and pollinating insects, exhibit positive phototaxis toward artificial lights in agricultural areas, forests, greenhouses, and granaries at night [143]. For example, two major tea pests, *Ectropis obliqua* and *Empoasca onukii*, have strong sensitivity to 385 and 420 nm wavelength light, and their dominant natural insect enemies prefer a wavelength of 380 nm, so light traps with a combination of 385 and 420 nm wavelengths emitted with light-emitting diodes (LEDs) have been used to control tea insect pests. Indeed, LED light traps trapped more tea pests and fewer natural enemies than control traps fitted with a fluorescent lamp [144]. Cavaletto et al. [142] suggested that non-flower-visiting longhorn beetles were more attracted by dark and long wavelength-dominated colors, such as red and brown. Color is a characteristic that is determined by differing qualities of light being reflected or emitted. However, reports on the phototactic behavior of longhorn beetles are very rare. The maximum number of individuals captured was 12.5 beetles per trap per week when using only sex pheromones in an *Euscepes postfasciatus* (Fairmaire) trapping program, whereas the trapped number significantly increased when green LEDs were added to sex pheromones [145]. The phototactic behavior of agricultural pests has been well recognized, and traps equipped with specific light sources have made great contributions to IPM programs [143,146]. A previous study showed that the percentages of foraging and moving behavior of *A. glabripennis* at night were significantly higher than those during the day [147], suggesting that a light-trapping strategy may be used to monitor and manage the population density. Therefore, the phototactic behavior of longhorn beetles toward artificial light needs further investigation. Light traps baited with semiochemicals may improve the limited attractiveness of single chemical cues in the field. 

In addition, insects usually have three visual opsin proteins (UV, SW, and LW), which form photopigments that are maximally sensitive to ultraviolet, blue, and green wavelengths [54,148,149,150]. Molecular evidence suggests that opsins, which detect blue wavelengths, were lost approximately 300 million years ago in many beetle lineages, including those containing the ALB, diving beetles (*Thermonectus marmoratus*), and jewel beetles (Buprestidae) [151]. The opsin family is divided into visual and nonvisual opsin subfamilies [152]. We found that a bark-mimicking color (forest green, CMYK: 54, 8, 100, 30) enhanced the response of adult insects to odor cues from the cut branches of host plants [20]. However, little is known about the opsin types in ALBs. Research on visual opsin types and pathways of visual signals may also provide a theoretical basis for the improvement of ALB trapping and management strategies.

## Figures and Tables

**Table 1:** The attractive and repellent volatile semichemical compounds in the host or nonhost plants of ALB.

Attractive Effect	Type	Semichemical Compound	Number	Reference(s)
Attraction	Alcohols	Butanol, pentanol, linalool, *Cis*-3-hexen-1-ol	4	[64,65,66,67]
	Aldehyde	Nonanal	1	[67]
	Olefins	*β*-caryophyllene, *δ*-3-carene, *S*-*β*-pinene, *R*-*α*-pinene, camphene, *D*-limonene (attracts only females), phellandrene	7	[44,67,68,69]
	Ester	Ethyl acetate	1	[44]
Repellent	Alcohols	trans-2-hexenol, (*E*)-1-pentylene-3-ol	2	[25]
	Olefins	Heptylene, ocimene, myrcene, *β*-caryophyllene, *β*-pinene	5	[25,44,67,69,70]
	Ketones	Geranyl acetone	1	[25]
	Ester	3-Hexenyl acetate	1	[68,69]

**Table 2:** High expression tissue and optimal binding ligands of four recombinant odorant-binding proteins.

OBP	High Expression Tissue	Chemical Type(s)	Optimal Ligand(s)	Ki (Μm/L)	References
*Agla*OBP1	ML, FA, MA, FL ^&^	Host kairomone	*Cis*-3-Hexen-1-ol	5.88	[78,79,80]
			*Cis*-2-Hexen-1-ol	8.33	
			*α*-Pinene	9.07	
		Host kairomone and volatile pheromone	*β*-caryophyllene	7.47	
*Agla*OBP12	FA, MA, ML, FL	Host kairomone and volatile pheromone	*β*-caryophyllene	0.74	[77,80,81]
		Host kairomone	*Cis*-3-Hexenyl acetate	0.77	
			Dodecanal	0.82	
			1-tetradecanol	0.96	
		Volatile pheromone	*α*-Farnesol	1.03	
*Agla*OBP45	MA	Host kairomone	Benzyl alcohol	0.92	[79]
			2-Pentanol	0.96	
			*α*-Ocimene	1.02	
			*α*-Pinene	1.92	
		Long-range sex pheromone	Hexadecanal	0.87	
		Female contact and trail sex pheromone	(*Z*)-9-tricosene	2.02	
			(*Z*)-9-pentacosene	6.81	
*Agla*OBP46	MA, FA, ML, FL	Host kairomone	1-Dodecanol	0.74	[79]
			Benzyl alcohol	0.82	
			2-Pentanol	2.12	
			(*+*)-longifolene	1.78	
			*D*-limonene	2.01	

Notes: MA—male antenna; FA—female antenna; ML—male leg; FL—female leg. ^&^: the sequence of high-expression tissue is arranged by the expression level. The Binding constants of recombinant *Agla*OBP 1, 12, 45, and 46 with *N*-phenyl-1-naphthylamine were 7.65, 3.25, 3.37, and 3.25 μM/L, respectively. Host kairomones and sex pheromones are classical based on the information presented in Table 1 and Table S4.

**Table 3:** The effects of sex pheromones and host kairomones on ALB traps in the green house and field.

Study	Chemical Matter	Number Captured	Interval Time	Site	Reference
1	(*-*)-linalool	3.00 ± 1.00	7 days	Green house	[68]
2	(*-*)-linalool + MP	F: 3.70 ± 3.20	7 days	Ningxia, China	[97]
		M: 1.70 ± 1.20	7 days		
3	Heptanal, nonanal, hexadecanal (1:7:1)	4.70 ± 2.20	7 days	Ningxia, China	[98]
4	Heptanal, nonanal, hexadecanal + HK	3.20 ± 1.20	7 days		
5	MP + PV (1:1)	2.25	7 days	Syracuse, NY, USA	[99]
6	PV (8:9:1)	1.10 ± 0.89	7 days	Cixi, China	[100]
7	MK	3.40 ± 1.10	7 days	Cixi, China	[101]
8	Brown trap + 2-pentanol	F: 1.52 ± 0.12	7 days	Hengshui, China	[102]
		M: 1.00 ± 0.09			
9	25 mg α-longipinene	0.90 ± 0.50	7 days	Huanyuan, China	[80]
	5 mg *α*-longipinene + pv	1.10 ± 0.50			

Notes: MP: male-produced pheromone (4-(n-Heptyloxy)butanol and 4-(n-Heptyloxy)butanal); HK: host kairomones, blend of *cis*-3-hexen-1-ol, camphene, *delta*-3-carene, linalool, and β-caryophyllene; PV: blend of (-)-linalool, β-caryophyllene and *cis*-3-hexen-1-ol; MK: male-produced pheromone + camphene + *cis*-3-hexen-1-ol + ocimene + *β*-caryophyllene; pv: linalool, linalool oxide, *cis*-3-hexen-1-ol, camphene, *β*-caryophyllene, and 3-carene; F: female, M: male. Data are presented as the mean ± SEM from the optimal trapping lure.

## Data Availability

The data supporting reported results can be found in the manuscript.

## Supplementary Materials

The following supporting information can be downloaded at: https://www.mdpi.com/article/10.3390/insects14030292/s1. Table S1: List of host plants of the ALB. Table S2: The categorization of host trees species for ALB in the literature. Table S3: Binding affinities of four recombinant *Agla*OBPs with different candidate ligands assessed by fluorescent competitive binding assays. Table S4: The diversity in sex pheromone composition of the ALB. PPT S1: Trap data shown in Table 3 from different references. References [6,17,18,21,22,23,24,25,26,27,28,29,30,31,32,33,34,35,36,37,38,39,40,41,42,43,44,45,46,47,48,49,50,51,52,53,68,77,79,80,81,95,96,97,98,99,100,102,104,105] are cited in the supplementary materials
