# Supplementary material for: A Review of the Host Plant Location and Recognition Mechanisms of Asian Longhorn Beetle
Source: Insects. 2023 Mar 17;14(3):292. doi: 10.3390/insects14030292 (PMC10054519; doi:10.3390/insects14030292)
Supplement: Supplementary file 1 [file insects-14-00292-s001.zip › Supplementary files S1-Table S1.docx]

Table S1. List of host plants of the ALB

| Serial number | Family | Genus | HS | Host species | Lifestyle | | | | Complete lifestyle | Sources | References |
| --- | --- | --- | --- | --- | --- | --- | --- | --- | --- | --- | --- |
|  |  |  |  |  | Feeding | Egg sites | Oviposition | Exit holes |  |  |  |
| 1 | Sapindaceae | *Acer* | Y | *Acer buergerianum* Miquel | Y | Y | Y | Y | Y | C | [21] |
| 2 |  |  | Y | *Acer campestre* L. | Y | Y | Y | Y | Y | E | [22] |
| 3 |  |  | Y | *Acer davidii* Franchet | Y | Y | Y | Y | Y | C | [23] |
| 4 |  |  | Y | *Acer × freemanii* ‘Autumn Blaze’ | Y | Y | Y | Y | Y | C | [24] |
| 5 |  |  | Y | *Acer ginnala* Maximowicz | Y | Y | Y | Y | Y | C, U | [21] |
| 6 |  |  | Y | *Acer grosseri* Pax | Y | Y | Y | Y | Y | C | [23] |
| 7 |  |  | Y | *Acer mono* Maximowicz | Y | Y | Y | Y | Y | C, U | [25-27] |
| 8 |  |  | Y | *Acer negundo* | Y | Y | Y | Y | Y | C, U, E | [22, 25-28] |
| 9 |  |  | Y | *Acer nigrum* F. | Y | Y | Y | Y | Y | U | [26] |
| 10 |  |  | Y | *Acer oliverianum* Pax | Y | Y | Y | Y | Y | C | [29] |
| 11 |  |  | Y | *Acer palmatum* Thunberg | Y | Y | Y | Y | Y | C, E | [21, 26] |
| 12 |  |  | Y | *Acer palmatum* cv. *Apropurpureum* | Y | Y | Y | Y | Y | C | [26, 29] |
| 13 |  |  | Y | *Acer pictum* subsp. mono (Maxim.) Ohashi | Y | Y | Y | Y | Y | K | [30] |
| 14 |  |  | Y | *Acer pictum* var. truncatum (Bunge) C. S. Chang | Y | Y | Y | Y | Y | K | [30] |
| 15 |  |  | Y | *Acer platanoides* L. | Y | Y | Y | Y | Y | U | [28] |
| 16 |  |  | Y | *Acer platanoides* ‘Royal Red’ | Y | Y | Y | Y | Y | C | [24, 25] |
| 17 |  |  | Y | *Acer platanoides* ‘Superform’ | Y | Y | Y | Y | Y | C | [24] |
| 18 |  |  | Y | *Acer pseudoplatanus* L. | Y | Y | Y | Y | Y | U, E | [22, 26, 28] |
| 19 |  |  | Y | *Acer rubrum* | Y | Y | Y | Y | Y | E | [26] |
| 20 |  |  | Y | *Acer rubrum* ‘Red Sunset’ | Y | Y | Y | Y | Y | C | [24] |
| **Continued** | | | | | | | | | | | |
| Serial number | Family | Genus | HS | Host species | Lifestyle | | | | Complete lifestyle | Sources | References |
|  |  |  |  |  | Feeding | Egg sites | Oviposition | Exit holes |  |  |  |
| 21 |  |  | Y | *Acer saccharinum* L. | Y | Y | Y | Y | Y | U, E | [26, 28] |
| 22 | Sapindaceae |  | Y | *Acer saccharum* Marshall | Y | Y | Y | Y | Y | C, U | [26, 31] |
| 23 |  |  | Y | *Acer tataricum* subsp. ginnala | Y | Y | Y | Y | Y | C | [31, 32] |
| 24 |  |  | Y | *Acer tegmentosum* Maxim. | Y | Y | Y | Y | Y | K | [30] |
| 25 |  |  | Y | *Acer truncatum* Bunge | Y | Y | Y | Y | Y | C | [25, 26] |
| 26 |  |  | Y | *Acer truncatum* × Norwegian Sunset | Y | Y | Y | Y | Y | C | [24] |
| 27 |  |  | Y | *Acer truncatum*×Pacific Sunset | Y | Y | Y | Y | Y | C | [24] |
| 28 | Hippocastanaceae | *Aesculus* | Y | *Aesculus carnea* J. Zeyh. | Y | Y | Y | Y | Y | E | [22] |
| 29 |  |  | Y | *Aesculus chinensis* Bunge | Y | Y | Y | Y | Y | U | [26] |
| 30 |  |  | Y | *Aesculus x carnea* Hayne | Y | Y | Y | Y | Y | E | [26] |
| 31 |  |  | Y | *Aesculus flava* Solander | Y | Y | Y | Y | Y | U | [26] |
| 32 |  |  | Y | *Aesculus glabra* Willdenow | Y | Y | Y | Y | Y | U | [26] |
| 33 |  |  | Y | *Aesculus hippocastanum* | Y | Y | Y | Y | Y | U, E | [6, 26, 28] |
| 34 | Salicaceae | *Populus* | Y | *Populus alba* and three hybrids | Y | Y | Y | Y | Y | C | [21, 26, 27] |
| 35 |  |  |  | *Populus alba* L. var. *pyramidalis* | Y | Y | Y | Y | Y | C | [21, 26] |
| 36 |  |  | Y | *Populus alba*× *Populus berolinensis* | Y | Y | Y | Y | Y | C | [31] |
| 37 |  |  |  | *Populus alba × Populus bolleana* | Y | Y | Y | Y | Y | C | [21, 27] |
| 38 |  |  |  | *Populualba* Var. *Payrarridalis* × *P.alba* | Y | Y | Y | Y | Y | C | [23] |
| 39 |  |  |  | *Populus alba* *× P. tomentosa* | Y | Y | Y | Y | Y | C | [33] |
| 40 |  |  |  | *Populus babylonica* L. | Y | Y | Y | Y | Y | C | [26] |
| 41 |  |  |  | *Populus balsamifera* L. |  | Y |  |  |  | U | [26] |
| **Continued** | | | | | | | | | | | |
| Serial number | Family | Genus | HS | Host species | Lifestyle | | | | Complete lifestyle | Sources | References |
|  |  |  |  |  | Feeding | Egg sites | Oviposition | Exit holes |  |  |  |
| 42 |  |  | Y | *Populus × beijingensis* W. Y. Hsu | Y | Y | Y | Y | Y | C | [21] |
| 43 |  |  |  | *Populus × berolinensis* Dippel | Y | Y | Y | Y | Y | C | [31, 34] |
| 44 |  |  |  | *Populus × russki* | Y | Y | Y | Y | Y | C | [34] |
| 45 |  |  | Y | *Populus × canadensis* Moench | Y | Y | Y | Y | Y | C | [21, 27] |
| 46 |  |  |  | *Populus × canadensis* ‘Serotina’ | Y | Y | Y | Y | Y | E | [22] |
| 47 |  |  | Y | *Populus canadensis × euramericana* cv. I-58 | Y | Y | Y | Y | Y | C | [21] |
| 48 |  |  | Y | *Populus canadensis × euramericana* cv. I-214 | Y | Y | Y | Y | Y | C | [21] |
| 49 |  |  |  | *Populus canadensis × euramericana* cv. Jiqin-1 | Y | Y | Y | Y | Y | C | [21, 34] |
| 50 |  |  |  | *Populus canadensis × euramericana* cv. Jiqin-2 | Y | Y | Y | Y | Y | C | [21, 34] |
| 51 |  |  | Y | *Populus canadensis × euramericana* cv. Robusta | Y | Y | Y | Y | Y | C | [21] |
| 52 |  |  | Y | *Populus cathayana* Rehder | Y | Y | Y | Y | Y | C | [21, 27] |
| 53 |  |  |  | *Populus charbinensis* C. Wang et Skvortsov | Y | Y | Y | Y | Y | C | [31, 32] |
| 54 |  |  |  | *Populus charbinensis* *×* xiaohei | Y | Y | Y | Y | Y | C | [32] |
| 55 |  |  | Y | *Populus × dakuanensis* Hsu | Y | Y | Y | Y | Y | C | [21] |
| 56 |  |  | Y | *Populus × dakuanensis* Hsu *× p. nigra* cv. ‘Italica’ | Y | Y | Y | Y | Y | C | [23] |
| 57 |  |  |  | *Populus deltides* ‘Harvord’ ex I-63/51 | Y | Y | Y | Y | Y | C | [21, 23] |
| 58 |  |  |  | *Populus deltoides* × *(P. therstina × P. nigra)* | Y | Y | Y | Y | Y | C | [31] |
| 59 |  |  | Y | *Populus deltoides* var. Brangarsi | Y | Y | Y | Y | Y | C | [34] |
| 60 |  |  |  | *Populus deltoides* W. Bartram ex Marshall | Y | Y | Y | Y | Y | U | [26] |
| 61 |  |  | Y | *Populus deltoide*s cv. I-45/51/63 | Y | Y | Y | Y | Y | C | [23, 33] |
| 62 |  |  |  | *Populus deltoide*s cv. I-55/69 | Y | Y | Y | Y | Y | C | [23] |
| 63 |  |  |  | *Populus deltoide*s ‘Nankang’ | Y | Y | Y | Y | Y | C | [34] |
| 64 |  |  | Y | *Populus deltoide*s cv. ‘Shanhaiguanensis’ | Y | Y | Y | Y | Y | C | [35] |
| **Continued** | | | | | | | | | | | |
| Serial number | Family | Genus | HS | Host species | Lifestyle | | | | Complete lifestyle | Sources | References |
|  |  |  |  |  | Feeding | Egg sites | Oviposition | Exit holes |  |  |  |
| 65 |  |  |  | *Populus eugenei* Dode | Y | Y | Y | Y | Y | C | [22] |
| 66 |  |  |  | *Populus euphratica* Oliver | Y | Y | Y | Y | Y | C | [21] |
| 67 |  |  |  | *Populus × euramericana* (Dode) Guinier | Y | Y | Y | Y | Y | C | [21] |
| 68 |  |  | Y | *Populus euramevicana* cv. I-214 | Y | Y | Y | Y | Y | C | [33] |
| 69 |  |  |  | *Populus euramevicana* (Poda cv. ‘I-214’ *×P. pekinensis* | Y | Y | Y | Y | Y | C | [23, 33] |
| 70 |  |  | Y | *Populus euramevicana* cv. Polska-15A | Y | Y | Y | Y | Y | C | [23, 33] |
| 71 |  |  |  | *Populus euramericana* CL. ‘San. Martina’ | Y | Y | Y | Y | Y | C | [21] |
| 71 |  |  | Y | *Populus euramericana* cv. ‘Eugene’ | Y | Y | Y | Y | Y | C | [23, 33] |
| 72 |  |  |  | *Populus × euramericana* (Dode) Guiner CL. ‘zhonglin46’ | Y | Y | Y | Y | Y | C | [36] |
| 73 |  |  | Y | *Populus × euramericana* Guinier cv. ‘Sacrau 79’ | Y | Y | Y | Y | Y | C, E | [23, 26] |
| 74 |  |  | Y | *Populus euramericana* cv. ‘Bellini’ | Y | Y | Y | Y | Y | C | [34] |
| 75 |  |  | Y | *Populus euramericana* cv. ‘Guardi’ | Y | Y | Y | Y | Y | C | [34] |
| 76 |  |  | Y | *Populus euramericana* Luisa Avanzo | Y | Y | Y | Y | Y | C | [34, 36] |
| 77 |  |  |  | *Populus euramericana* cv. ‘Ieipuig’ | Y | Y | Y | Y | Y | C | [33] |
| 78 |  |  | Y | *Populus gansuensis* Z. Wang et H.L. Yang | Y | Y | Y | Y | Y | C | [21, 27] |
| 79 |  |  |  | *Populus grandidentata* Michaux |  |  |  |  |  | U | [26] |
| 80 |  |  |  | *Populus hopeiensis* Hu et Chow and 1 hybrid | Y | Y | Y | Y | Y | C | [21, 27] |
| 81 |  |  | Y | *Populus hybrid* | Y | Y | Y | Y | Y | C | [27] |
| 82 |  |  |  | *Populus lasiocarpa* Oliver | Y | Y | Y | Y | Y | C | [34] |
| 83 |  |  | Y | *Populus maximowiczii* A. Henry | Y | Y | Y | Y | Y | C, K | [21] |
| 84 |  |  | Y | *Populus nigra* L. var. *italica* (Moench) Koehne | Y | Y | Y | Y | Y | C | [21, 27, 34] |
| 85 |  |  | Y | *Populus nigra* var. *pyramidalis* | Y | Y | Y | Y | Y | C | [34] |
| **Continued** | | | | | | | | | | | |
| Serial number | Family | Genus | HS | Host species | Lifestyle | | | | Complete lifestyle | Sources | References |
|  |  |  |  |  | Feeding | Egg sites | Oviposition | Exit holes |  |  |  |
| 86 |  |  |  | *Populus nigra* var. ‘*Thevestina*’ | Y | Y | Y | Y | Y | C | [27, 34] |
| 87 |  |  |  | *Populus nigra × p. trichocarpa* | Y | Y | Y | Y | Y | C | [33] |
| 88 |  |  |  | *Populus nigra × P. simonii* | Y | Y | Y | Y | Y | C | [31, 34] |
| 89 |  |  | Y | *Populus nigra* L. var. *thevestina* (Dode) Bean | Y | Y | Y | Y | Y | C | [21, 27] |
| 90 |  |  |  | *Populus nigra* L. cv. ‘blane de garonne’ | Y | Y | Y | Y | Y | C | [33] |
| 91 |  |  | Y | *Populus nigra* L. var. *italic* (Moench) Koehne | Y | Y | Y | Y | Y | C | [27] |
| 92 |  |  |  | *Populus pekinensis* L.Henry | Y | Y | Y | Y | Y | C | [26] |
| 93 |  |  |  | *Populus pordomii* Rehd. | Y | Y | Y | Y | Y | C | [23] |
| 94 |  |  |  | *Populus pseudoglauca* Z.Wang *et* P.Y.Fu | Y | Y | Y | Y | Y | C | [34] |
| 95 |  |  |  | *Populus pseudosimonii* Kitagawa | Y | Y | Y | Y | Y | C | [21] |
| 96 |  |  | Y | *Populus purdomii* Rehder | Y | Y | Y | Y | Y | C | [23] |
| 97 |  |  |  | *Populus* ‘SANBEI’ No. 1 | Y | Y | Y | Y | Y | C | [27] |
| 98 |  |  |  | *Populus* × *simopyramdalis* | Y | Y | Y | Y | Y | C | [26] |
| 99 |  |  |  | *Populus simonii* Carrière | Y | Y | Y | Y | Y | C | [21, 27] |
| 100 |  |  |  | *Populus simonii × P. nigra ‘Pyramidalis’ ‘*Baichensis’ | Y | Y | Y | Y | Y | C | [26, 34] |
| 101 |  |  |  | *Populus simonii × P. nigra ‘Pyramidalis’ ‘*Taiqing’ | Y | Y | Y | Y | Y | C | [34] |
| 102 |  |  |  | *Populus simonii × P. nigra ‘Pyramidalis’ ‘*Italica’ | Y | Y | Y | Y | Y | C | [34] |
| 103 |  |  | Y | *Populus simonii × Populus pyramibalis* cv. Opera | Y | Y | Y | Y | Y | C | [21] |
| 104 |  |  |  | *Populus stalinetz* Aigeiros | Y | Y | Y | Y | Y | C | [34] |
| 105 |  |  |  | *Populus s. euphratica* Oliv*. P. alba L.* var*. pyramdalis* Bunge | Y | Y | Y | Y | Y | C | [27] |
| **Continued** | | | | | | | | | | | |
| Serial number | Family | Genus | HS | Host species | Lifestyle | | | | Complete lifestyle | Sources | References |
|  |  |  |  |  | Feeding | Egg sites | Oviposition | Exit holes |  |  |  |
| 106 |  |  |  | *Populus tomentosa* Carrière | Y | Y | Y | Y | Y | C | [27] |
| 107 |  |  |  | *Populus tomentosa* cv. ‘Hopeinica’ |  |  | Y |  |  | C | [21] |
| 108 |  |  |  | *Populus tomentosa* Carrière var tianshui | Y | Y | Y | Y | Y | C | [23, 29] |
| 109 |  |  |  | *Populus tomentosa* Carrière var. truncata Y.C.Fu et C.H.Wang | Y | Y | Y | Y | Y | C | [23] |
| 110 |  |  |  | *Populus tremuloides* Michaux |  | Y | Y | Y | Y | U | [26] |
| 111 |  |  | Y | *Populus × xiaohei* T. S. Hwang *et* Liang | Y | Y | Y | Y | Y | C | [21, 31] |
| 112 |  |  |  | *Populus × xiaohei* ‘Helin-1’ | Y | Y | Y | Y | Y | C | [34] |
| 113 |  |  | Y | *Populus × xiaozhuanica* W.Y. Hsu *et* Liang cv.‘Baicheng-2’ | Y | Y | Y | Y | Y | C | [21] |
| 114 |  |  | Y | *P**opulus × xiaozhuanica* W.Y. Hsu *et* Liang cv.‘Balizhuangyang’ | Y | Y | Y | Y | Y | C | [38] |
| 115 |  |  | Y | *Populus × xiaozhuanica* cv. ‘*Shuangyangensis*’ | Y | Y | Y | Y | Y | C | [21] |
| 116 |  |  | Y | *Populus × xiaozhuanica* cv. ‘Opera’ | Y | Y | Y | Y | Y | C | [34] |
| 117 |  |  | Y | *Populus × xiaozhuanica* cv. ‘Popularis’ | Y | Y | Y | Y | Y | C | [34] |
| 118 |  |  | Y | *Populus × xiaozhuanica* | Y | Y | Y | Y | Y | C | [21] |
| 119 |  |  |  | *Populus ussuriensis* Komarov | Y | Y | Y | Y | Y | C | [27] |
| 120 |  | *Salix* | Y | *Salix alba* L. | Y | Y | Y | Y | Y | U | [26] |
| 121 |  |  | Y | *Salix × aureo-pendula* | Y | Y | Y | Y | Y | C | [26, 27] |
| 122 |  |  | Y | *Salix aurita* L. | Y | Y | Y | Y | Y | C, E | [21] |
| **Continued** | | | | | | | | | | | |
| Serial number | Family | Genus | HS | Host species | Lifestyle | | | | Complete lifestyle | Sources | References |
|  |  |  |  |  | Feeding | Egg sites | Oviposition | Exit holes |  |  |  |
| 123 |  |  | Y | *Salix babylonica* | Y | Y | Y | Y | Y | C | [37] |
| 124 |  |  | Y | *Salix babylonica ×Salix alba f. vitellina* | Y | Y | Y | Y | Y | C | [21] |
| 125 |  |  | Y | *Salix caprea* L. | Y | Y | Y | Y | Y | U, E | [26] |
| 126 |  |  | Y | *Salix cinerea* L. | Y | Y | Y | Y | Y | E | [26] |
| 127 |  |  | Y | *Salix discolor* Muhlenberg | Y | Y | Y | Y | Y | E | [26] |
| 128 |  |  | Y | *Salix fragilis* L. | Y | Y | Y | Y | Y | U, E | [22, 26] |
| 129 |  |  | Y | *Salix gordeivii* Chang *et* SkV. | Y | Y | Y | Y | Y | C | [40] |
| 130 |  |  | Y | *Salix ohsidare* | Y | Y | Y | Y | Y | C | [41] |
| 131 |  |  | Y | *Salix paraplesia* Schneid. var. *subintegra* C.Wang e | Y | Y | Y | Y | Y | C | [39] |
| 132 |  |  | Y | *Salix matsudana* Koidz | Y | Y | Y | Y | Y | C | [28, 42] |
| 133 |  |  | Y | *Salix matsudana f. lobato-glandulosa* | Y | Y | Y | Y | Y | C | [43] |
| 134 |  |  | Y | *Salix matsudana f. tortuosa* | Y | Y | Y | Y | Y | C | [21] |
| 135 |  |  | Y | *Salix matsudana* var. *matsudana* f. *umbraculifera* Rehd. | Y | Y | Y | Y | Y | C | [21] |
| 136 |  |  | Y | *Salix matsudana f. pendula* | Y | Y | Y | Y | Y | C | [21] |
| 137 |  |  | Y | *Salix matsudana sp.* | Y | Y | Y | Y | Y | C | [44] |
| 138 |  |  | Y | *Salix nankingensis* C. Wang et S. L. Tung | Y | Y | Y | Y | Y | C | [21] |
| 139 |  |  |  | *Salix nigra* Marshall |  |  | Y |  |  | U | [26] |
| 140 | Ulmaceae | *Ulmus* |  | *Ulmus Americana* L. |  |  | Y | Y | Y | U | [26] |
| 141 |  |  | Y | *Ulmus campestris* L. | Y | Y | Y | Y | Y | C | [23] |
| 142 |  |  | Y | *Ulmus davidiana* Planch var. japonica (Rehd.) Nakai | Y | Y | Y | Y | Y | C | [27] |
| 143 |  |  |  | *Ulmus densa* | Y | Y | Y | Y | Y | C | [45] |
| 144 |  |  | Y | *Ulmus glabra* | Y | Y | Y | Y | Y | E | [18] |
| **Continued** | | | | | | | | | | | |
| Serial number | Family | Genus | HS | Host species | Lifestyle | | | | Complete lifestyle | Sources | References |
|  |  |  |  |  | Feeding | Egg sites | Oviposition | Exit holes |  |  |  |
| 145 |  |  | Y | *Ulmus laciniata* (Trautv.) Matruchot | Y | Y | Y | Y | Y | C | [21] |
| 146 |  |  |  | *Ulmus macrocarpa* Hance | Y | Y | Y | Y | Y | C | [27] |
| 147 |  |  |  | *Ulmus minor* | Y | Y | Y | Y | Y | C | [45] |
| 148 |  |  | Y | *Ulmus parvifolia* Jacquin | Y | Y | Y | Y | Y | U, K | [28, 30] |
| 149 |  |  | Y | *Ulmus pumila* L. | Y | Y | Y | Y | Y | C | [27] |
| 150 |  |  | Y | *Ulmus pumila* L. *f. tenue* S. Y. Wang | Y | Y | Y | Y | Y | C | [21] |
| 151 |  |  | Y | *Ulmus pumila* var. *pendula* | Y | Y | Y | Y | Y | C | [21] |
| 152 | Fabaceae | *Albizia* |  | *Albizia julibrissin* Durazzini |  |  | Y | Y | Y | U, E | [26] |
| 153 |  | *Alnus* |  | *Alnus incana* (L.) Moench | Y | Y | Y | Y | Y | C | [6] |
| 154 |  |  |  | *Alnus rubra* Bongard |  |  |  |  |  | E | [26] |
| 155 |  | *Amorpha* |  | *Amorpha fruticosa* L. |  |  | Y |  |  | C | [21, 26] |
| 156 |  | *Armeniaca* |  | *Armeniaca sibiria* (L.) Lam | Y |  |  |  |  | C | [27] |
| 157 | Betulaceae | *Betula* |  | *Betula nigra* L. |  |  | Y | Y | Y | U | [26] |
| 158 |  |  |  | *Betula papyrifera* Marshall |  |  | Y | Y | Y | U | [26] |
| 159 |  |  | Y | *Betula pendula* Roth | Y | Y | Y | Y | Y | E | [22, 26] |
| 160 |  |  | Y | *Betula platyphylla* Sukaczev | Y | Y | Y | Y | Y | C | [6, 26, 46, 47] |
| 161 |  |  |  | *Betula populifolia* Marshall |  |  | Y | Y | Y | U | [26] |
| 162 |  |  |  | *Betula pubescens* Ehrhart |  |  | Y | Y | Y | U | [26] |
| 163 | Fabaceae | *Cajanus* |  | *Cajanus indicus* Spreng |  |  |  |  |  | K | [30] |
| 164 | Betulaceae | *Carpinus* |  | *Carpinus betulus* L. | Y | Y | Y | Y | Y | E | [22, 26] |
| 165 |  |  |  | *Carpinus caroliniana* Walter |  |  | Y |  |  | E | [26] |
| **Continued** | | | | | | | | | | | |
| Serial number | Family | Genus | HS | Host species | Lifestyle | | | | Complete lifestyle | Sources | References |
|  |  |  |  |  | Feeding | Egg sites | Oviposition | Exit holes |  |  |  |
| 166 | Juglandanceae | *Carya* | Y | *Carya cathayensis* Sargent | Y | Y | Y | Y | Y | C | [48] |
| 167 |  | *Casuarina* |  | *Casuarina equisetifolia* | Y | Y | Y | Y | Y | C | [27] |
| 168 | Ulmaceae | *Celtis* |  | *Celtis sinensis* Persoon | Y | Y | Y |  |  | C | [23, 26] |
| 169 | Cercidiphyllaceae | *Cercidiphyllum* |  | *Cercidiphyllum japonicum* Siebold & Zuccarini *ex* J.J. Hoffman & J.H. Schultz bis | Y | Y | Y | Y | Y | C, U | [23, 26] |
| 170 | Lauraceae | *Cinnamomum* |  | *Cinnamomum camphora* | Y | Y | Y | Y | Y | C | [24] |
| 171 |  | *Corylus* |  | *Corylus* L. |  |  | Y |  |  | E | [26] |
| 172 | Rosaceae | *Crataegus* | Y | *Crataegus monogyna* Jacquin | Y | Y | Y | Y | Y | C | [26] |
| 173 |  | *Crataegus* | Y | *Crataegus pinnatifida* Bge. | Y | Y | Y | Y | Y | C | [27] |
| 174 | Elaeagnaceae | *Elaeagnus* |  | *Elaeagnus angustifolia* L. | Y | Y | Y | Y | Y | C, U | [26, 49] |
| 175 | Fagaceae | *Fagus* |  | *Fagus sylvatica* L. and two varieties | Y | Y | Y | Y | Y | E | [26] |
| 176 | Malvaceae | *Firmiana* |  | *Firmiana simplex* (L.) W. Wight |  |  | Y |  |  |  | [21] |
| 177 | Oleaceae | *Fraxinus* |  | *Fraxinus Americana L.* |  |  | Y |  |  | C | [26] |
| 178 |  |  |  | *Fraxinus chinensis* Roxburgh | Y | Y | Y | Y | Y | C | [27, 43] |
| 179 |  |  |  | *Fraxinus chinensis Roxb.* Var. *Rhynchophylla* (Hance) Hemsl | Y | Y | Y | Y | Y | C | [27] |
| 180 |  |  |  | *Fraxinus excelsior* L. |  |  | Y |  |  | U | [50] |
| 181 |  |  |  | *Fraxinus pennsylvanica* Marsh | Y | Y | Y | Y | Y | U | [26, 51] |
| 182 |  |  |  | *Gleditsia triacanthos* | Y | Y | Y | Y | Y | U | [52] |
| 183 | Hibisceae | *Hibiscus* |  | *Hibiscus syriacus* L. | Y | Y | Y |  |  | C | [23] |
| 184 | Elaeagnaceae | *Hippophae* |  | *Hippophae rhamnoides* L. | Y | Y | Y | Y | Y | C | [23] |
| **Continued** | | | | | | | | | | | |
| Serial number | Family | Genus | HS | Host species | Lifestyle | | | | Complete lifestyle | Sources | References |
|  |  |  |  |  | Feeding | Egg sites | Oviposition | Exit holes |  |  |  |
| 185 | Sapindaceae | *Koelreuteria* |  | *Koelreuteria paniculata* | Y | Y | Y | Y | Y | C | [6, 26] |
| 186 |  |  |  | *Koelreuteria bipinnata* | Y |  |  |  |  | C | [21] |
| 187 | Oleaceae | *Ligustrum* |  | *Ligustrum lucidum* W.T. Aiton | Y |  |  |  |  | C | [29] |
| 188 | Rosaceae | *Malus* |  | *Malus baccata* (L.) Borkh | Y |  |  |  |  | C | [27] |
| 189 |  |  |  | *Malus pumila* Miller= *M. domestica* | Y | Y | Y | Y | Y | C, E | [6, 26] |
| 190 |  |  |  | *Malus spectabilis* (Aiton) Borkhausen |  |  | Y |  |  | C | [21, 26] |
| 191 | Meliaceae | *Melia* |  | *Melia azedarach* L. | Y | Y | Y |  |  | C | [41] |
| 192 | Moraceae | *Morus* |  | *Morus alba* | Y |  | Y |  |  | C | [27, 49] |
| 193 | Rosaceae | *Padus* |  | *Padus racemose* | Y | Y | Y | Y | Y | C | [27] |
| 194 | Platanaceae | *Platanus* |  | *Platanus acerifolia* Willdenow | Y | Y | Y | Y | Y | C, U | [21] |
| 195 |  |  |  | *Platanus occidentalis* L. | Y | Y | Y | Y | Y | C, U | [6] |
| 196 |  |  |  | *Platanus orientalis* L. | Y | Y | Y | Y | Y | C | [23, 29] |
| 197 | Rosaceae | *Pruns* |  | *Prunus armeniaca* L. |  | Y |  |  |  | C | [21, 26] |
| 198 |  |  |  | *Prunus persica* F. Duplex | Y |  |  |  |  | C | [29] |
| 199 |  |  |  | *Prunus salicina* Lindley |  | Y |  |  |  | E | [26] |
| 200 |  | *Pyrus* |  | *Pyrus bretschneideri* Rehder |  | Y |  |  |  | C | [21, 26] |
| 201 |  |  |  | *Pyrus betulaefolia* Bunge |  | Y |  |  |  | C | [21, 26] |
| 202 | Fagaceae | *Quercus* |  | *Quercus mongolica* Fisch. ex Ledeb | Y |  |  |  |  | C | [31] |
| 203 |  |  |  | *Quercus rubra* L. | Y | Y | Y | Y | Y | U | [17, 26] |
| 204 | Leguminosae | *Robinia* |  | *Robinia pseudoacacia* | Y |  |  |  |  | C | [29] |
| 205 | Tamaricaceae | *Tamarix* |  | *Tamarix chinensis* Loureiro | Y |  |  |  |  | C | [21] |
| 206 | Tilioideae | *Tilia* |  | *Tilia cordata* Miller |  | Y | Y |  |  | U | [26] |
| **Continued** | | | | | | | | | | | |
| Serial number | Family | Genus | HS | Host species or cultivars | Lifestyle | | | | Complete lifestyle | Sources | References |
|  |  |  |  |  | Feeding | Egg sites | Oviposition | Exit holes |  |  |  |
| 207 |  |  |  | *Tilia mongolica* Maximowicz | Y | Y | Y |  |  | C | [53] |
| 208 | Meliaceae | *Toona* |  | *Toona sinensis* (Juss.) M.Roemer | Y |  |  |  |  | C | [29] |
| 209 | Sapindaceae | *Xanthoceras* |  | *Xanthoceras sorbifolia* Bunge | Y | Y | Y | Y | Y | C | [27, 31] |

Notes: 1, some trees species were recorded as host plant of ALB, but feeding, oviposition or exit holes were found in the reference, for example, *Cajanus indicus* Spreng and *Alnus rubra* Bongard (yellow column); 2, The family and order which the trees species belong were checked in the websites (<https://encyclopedia.thefreedictionary.com/>); 3, HS indicated trees species on which ALB has been reported to complete its life cycle (from oviposition to emergence of new beetles), and it is also highly sensitive or very good host plant were recorded.

References

6. Van der Gaag, D.J.; Loomans, A.J.M. Host plants of *Anoplophora glabripennis*, a review. *EPPO Bulletin* **2014**, *44*, 518-528, doi:10.1111/epp.12151.

18. Faccoli, M.; Favaro, R. Host preference and host colonization of the Asian long-horned beetle, *Anoplophora glabripennis* (Coleoptera Cerambycidae), in Southern Europe. *Bull Entomol. Res.* **2016**, *106*, 359-367.

21. Wang, Z.G.; Huang, D.Z.; Yan, J.J. The sequence choice of the main tree species to *Anoplophora glabripennis* in northern part of China. *Journal of Agricultural University of Hebei* **2009**, *32*, 62-68, doi:10.3969/j.issn.1000-1573.2009.06.013.

22. Straw, N.A.; Fielding, N.J.; Tilbury, C.; Williams, D.T.; Inward, D. Host plant selection and resource utilisation by Asian longhorn beetle *Anoplophora glabripennis* (Coleoptera: Cerambycidae) in southern England. *Forestry* **2015**, *88*, 84-95, doi:10.1093/forestry/cpu037.

23. Gao, H.Z.; Yang, X.Y.; Wei, J.N.; Lang, X.R. An Investigation on the resisance of major forestation species to *Anoplophora glabripennis* and *A. nobilis*. *Journal of Northwest Forestry College* **1997**, *12*, 42-46.

24. Chen, B.Y.; Liao, Y.; Zhu, L. The occurrence and prevention and control techniques discussion of *Anoplophora glabropennis* on Camphor trees in nursery. *China Plant Protection* **2008**, *28*, 30-31.

25. Zhang, F.; Jin, Y.; Chen, H.; Wu, X. The selectivity mechanism of *Anoplphora glabripennis* on four different species of maples. *Acta Ecologica Sinica* **2006**, *26*, 870-877.

26. Meng, P.S.; Hoover, K.; Keena, M.A. Asian Longhorned Beetle (Coleoptera: Cerambycidae), an introduced pest of *Maple* and other hardwood trees in North America and Europe. *J. Integ. Pest Manag.* **2015**, *6*, 84-88, doi:10.1093/jipm/pmv003.

27. Wang, Z.; Yuan, K.Y.; Wang, X.M. Investigation of damages and annual life history of *Anoplophora glabripennis* in Baotou in the Inner Mongolia autonomous region. *Journal of Inner Mongolia Agricultural University (Natural Science Edition)* **2016**, *37*, 87-95.

28. An, Y.L. The occurrence of Asian longhorned beetle and its analysis in USA. *China Commodity Inspection* **1999**, 9-10.

29. Wang, F.; Zhou, J.; Yang, X. Study on the relationship between the behavior of selecting host of *Anoplophora nobilis* and host resistance on this beetle in mixed forest. *Scientia Silvae Sinicae* **2000**, *36*, 58-65.

30. Lim, J.; Jung, S.Y.; Lim, J.S.; Jang, J.; Kim, K.M.; Lee, Y.M.; Lee, B.W. A review of host plants of Cerambycidae (Coleoptera: Chrysomeloidea) with new host records for fourteen Cerambycids, including the Asian longhorn beetle (*Anoplophora glabripennis* Motschulsky), in Korea. *Korean Journal of Applied Entomology* **2014**, *53*, 111-133.

31. Guan, Z. The occurrence regulation of *Anoplophora glabripennis* in the Harbin. Master thesis, University of Heilongjiang, Harbing, 2010.

32. Jiang, W.N.; Liu, J.F.; Dong, X.W. Distribution and damage characteristics of *Anoplophora glabripennis* in Heilongjiang Province. *Protection Forest Science and Technology* **2012**, 71-72+77.

33. Yang, X.Y.; Yan, X.H.; Zhou, J.X. Resistance of populus spp. to *Anoplophora glabripennis*. *Journal of Northwest Forestry College* **1991**, *6*, 30-38.

34. Hu, J.; Angeli, S.; Schuetz, S.; Luo, Y.; Hajek, A.E. Ecology and management of exotic and endemic Asian longhorned beetle *Anoplophora glabripennis*. *Agr. Forest Entomol.* **2009**, *11*, 359-375, doi:10.1111/j.1461-9563.2009.00443.x.

35. Zhang, Y.; Huang, D.; Wang, Z.; Yan, Y.; Yin, J. Effects of secondary metabolites of poplar trees on the carboxylesterase and glutathione-S-transferase in *Anoplophora glabripennis*. *Scientia Silvae Sinicae* **2001**, *37*, 123-128.

36. Mu, Z.; Yin, R.; Qi, C.; Zhang, R.; Yu, J. A research for the occurrence of *Anoplophora glabripennis* Motschulsky in the poplar intensive management plantation. *Scientia Silvae Sinicae* **1999**, *35*, 148-152.

37. Tang, H.; Han, Y.; Ru, J.; Qu, M.; Wang, X. Evaluation of control effects on different measures against *Anoplophora glabripennis* by life table method. *Journal of Northwest Forestry University* **2020**, *35*, 101-107.

38. Wang, X.; Yuan, C.; Wang, J.; Fan, D.; Chu, X.; Jie, F.; An, B.; Ning, L. Study on the mechanism of inhibiting the hatching of eggs of *Anoplophora glabropennis* by I-69 Populus. In Proceedings of the Annual meeting of Entomological Society of China 2000; pp. 884-885.

39. Wang, Z.; Yan, J.; Liu, Y.; Tang, X.; Chen, Y. Investigation of *Anoplophora glabripennis* in Southern Tibet. *Journal of Northeast Forestry University* **2003**, *31*, 70-71.

40. Zhao, X.; Zhou, Z.; Guo, C.; Li, H.; Guo, S. Relationship between tannin content of willows poplar and damage of *Anoplophora glabripennis*. *Forest Investigation Design* **2011**, 73-75.

41. Zhu, N.; Zhang, D.Y.; Shen, H.X.; Hu, Q.; Fan, J.T. Oviposition preferences of *Anoplophora glabripennis* on the three host plants and composition analysis of host plant volatiles. *Journal of Zhejiang Agriculture and Forestry University* **2017**, *34*, 1059-1064.

42. Ma, W.; Zhou, Y.; Liu, Y.; Meng, Z.; Zhang, A.; Yan, S. Trapping efficacy of traps baited with four types of attractants for population evaluation of *Anoplophora glabripennis*. *Journal of Northeast Forestry University* **2021**, *49*, 120-123+130, doi:10.13759/j.cnki.dlxb.2021.05.021.

43. Yan, X.F.; Liu, Y.H. Host plant effects on weight, longevity, fecundity and hatching rate of *Anoplophora glabripennis*. *Chinese Agricultural Science Bulletin* **2012**, *28*, 52-56.

44. Fan, L.; Yan, S.; Sun, Z.; Meng, Z. EAG and behavioral responses of Asian longhorn beetle *Anoplophora glabripennis* (Coleoptera: Cerambycidae) to plant volatiles. *Chinese Journal of Ecology* **2013**, *32*, 142-148, doi:10.13292/j.1000-4890.2013.0055.

45. Bahatiguli, M. Investigation on the control of Asian longhorn beetle in Xinjiang by cutting tree crown. *Protection Forest Science and Technology* **2016**, 100-101+123 (In China), doi:10.13601/j.issn.1005-5215.2016.07.036.

46. Gao, R.; Wang, B.; Mastro, V.; Li, Y.; Wang, Y.; Yang, X. Infestation of Betula platyphylla by *Anoplophora glabripennis* and its control using insecticides. *Scientia Silvae Sinicae* **2009**, *45*, 163-166.

47. Qin, K. Study on the bionomics and control of technology of *Anoplophora glabripennis* in Harbin. Northeast Forestry University, 2009.

48. Du, H.; Wang, P.; Xu, H.; Zhang, W.; Wang, Z. EAG responses of Asian longhorn beetle *Anoplophora glabripennis* (Coleoptera: Cerambycidae) to volatiles of hickory. *Journal of Zhejiang Agricultural and Forset University* **2016**, *33*, 166-171.

49. Qiao, H.L.; Luo, Y.Q.; Feng, X.; Sun, J.H.; Han, X.W. The resistance of the main host-tree species to *Anoplophora glabripennis* in Xinjiang. *Chinese Bulletin of Entomology* **2007**, *44*, 660-664.

50. Turgeon, J.J.; Chuck, J.; Smith, M.T.; Mary, O.; Scarr, T.A.; Ben, G. Records of unsuccessful attack by *Anoplophora glabripennis* (Coleoptera: Cerambycidae) on broadleaf trees of questionable suitability in Canada. *Can. Entomol.* **2016**, *148*, 569-578.

51. Gu, Q.; Jia, R.; Li, H.; Guo, S.; Xu, T.; Lu, P.; Qiao, H. Host adaptability of *Anoplophora glabripennis* based on metabolomics and insect intestinal bacteria. *Journal of Plant Protection* **2022**, *49*, 1750-1763, doi:10.13802/j.cnki.zwbhxb.2022.2021067.

52. Bancroft, J.S.; Smith, M.T.; Tropp, C.J. Rapid test of the suitability of host-trees and the effects of larval history on *Anoplophora glabripennis* (Coleoptera: Cerambycidae). *J. Kansas Entomol. Society* **2002**, *75*, 308-316.

17. Morewood, W.D.; Neiner, P.R.; Mcneil, J.R.; Sellmer, J.C.; Hoover, K. Oviposition preference and larval performance of *Anoplophora glabripennis* (Coleoptera: Cerambycidae) in four eastern north American hardwood tree species. *Environ. Entomol.* **2003**, *32*, 1028-1034.

53. Tian, R.M.; Lu, Q.; Zhang, Y.F.; Sang, H. Induce and control of *Anoplophora glabripennis*. *Journal of Inner Mongolia Forestry Science and Technology* **2009**, *35*, 42-46.
