# Supplementary material for: A Review of the Host Plant Location and Recognition Mechanisms of Asian Longhorn Beetle
Source: Insects. 2023 Mar 17;14(3):292. doi: 10.3390/insects14030292 (PMC10054519; doi:10.3390/insects14030292)
Supplement: Supplementary file 1 [file insects-14-00292-s001.zip › Supplementary files S2-Table S2.docx]

Table S2 The categorization of host trees species for ALB in the literatures

| **Series** | **Order** | **Family** | **Genus** | **Species** | **Total category** | | | | **China** | | | | **North America** | | | | **Europe** | | | |
| --- | --- | --- | --- | --- | --- | --- | --- | --- | --- | --- | --- | --- | --- | --- | --- | --- | --- | --- | --- | --- |
|  |  |  |  |  | **HS** | **MS** | **PS** | **LS** | **HS** | **MS** | **PS** | **LS** | **HS** | **MS** | **PS** | **LS** | **HS** | **MS** | **PS** | **LS** |
| 1 | Sapindales | Sapindaceae | *Acer*^#^ | 27 | 27 |  |  |  | 18 |  |  |  | 8 |  |  |  | 6 |  |  |  |
| 2 |  | Hippocastanaceae | *Aesculus* | 6 | 6 |  |  |  |  |  |  |  | 4 |  |  |  | 3 |  |  |  |
| 3 | Malpighiales | Salicaceae | *Populus* | 86 | 36 | 47 |  | 3 | 36 | 45 |  | 1 |  | 2 |  | 2 | 1 | 1 |  |  |
| 4 |  |  | *Salix* | 20 | 19 |  |  | 1 | 14 |  |  |  | 3 |  |  | 1 | 5 |  |  |  |
| 5 | Rosales | Ulmaceae | *Ulmus* | 12 | 8 | 3 | 1 |  | 6 | 3 |  |  | 1 |  | 1 |  | 1 |  |  |  |
| 6 | Caryophyllales | Tamaricaceae | *Tamarix* | 1 |  |  |  | 1 |  |  |  | 1 |  |  |  |  |  |  |  |  |
| 7 | Fagales | Betulaceae | *Alnus* | 2 |  | 1 |  | 1 |  | 1 |  |  |  |  |  |  |  |  |  | 1 |
| 8 |  |  | *Betula* | 6 | 2 |  | 4 |  | 1 |  |  |  |  |  | 4 |  | 1 |  |  |  |
| 9 |  |  | *Carpinus* | 2 |  | 1 |  | 1 |  |  |  |  |  |  |  |  |  | 1 |  | 1 |
| 10 |  |  | *Corylus* | 1 |  |  |  | 1 |  |  |  |  |  |  |  |  |  |  |  | 1 |
| 11 |  | Casuarinaceae | *Casuarina* | 1 |  | 1 |  |  |  | 1 |  |  |  |  |  |  |  |  |  |  |
| 12 |  | Juglandanceae | *Carya* | 1 | 1 |  |  |  | 1 |  |  |  |  |  |  |  |  |  |  |  |
| 13 | Lamiales | Oleaceae | *Fraxinus* | 5 |  | 3 |  | 2 |  | 2 |  | 1 |  | 1 | 1 |  |  |  |  |  |
| 14 |  |  | *Ligustrum* | 1 |  |  |  | 1 |  |  |  | 1 |  |  |  |  |  |  |  |  |
| 15 | Laurales | Lauraceae | *Cinnamomum* | 1 |  | 1 |  |  |  | 1 |  |  |  |  |  |  |  |  |  |  |
| 16 | Malvales | Hibisceae | *Hibiscus* | 1 |  |  | 1 |  |  |  | 1 |  |  |  |  |  |  |  |  |  |
| 17 |  | Malvaceae | *Firmiana* | 1 |  |  |  | 1 |  |  |  | 1 |  |  |  |  |  |  |  |  |
| 18 |  |  | *Tilia* | 2 |  |  | 1 | 1 |  |  | 1 |  |  |  |  | 1 |  |  |  |  |
| 19 | Proteales | Platanaceae | *Platanus* | 3 |  | 3 |  |  |  | 3 |  |  |  | 2 |  |  |  |  |  |  |
| 20 | Rosales | Cannabaceae | *Celtis* | 1 |  |  | 1 |  |  |  | 1 |  |  |  |  |  |  |  |  |  |
| 21 |  | Elaeagnaceae | *Elaeagnus* | 1 |  | 1 |  |  |  | 1 |  |  |  | 1 |  |  |  |  |  |  |
| 22 |  |  | *Hippophae* | 1 |  | 1 |  |  |  | 1 |  |  |  |  |  |  |  |  |  |  |
| 23 |  | Fabaceae | *Albizia* | 1 |  |  | 1 |  |  |  |  |  |  |  | 1 |  |  |  | 1 |  |
| 24 |  |  | *Amorpha* | 1 |  |  |  | 1 |  |  |  | 1 |  |  |  |  |  |  |  |  |
| 25 |  |  | *Armeniaca* | 1 |  |  |  | 1 |  |  |  | 1 |  |  |  |  |  |  |  |  |
| 26 |  | Leguminosae | *Cajanus^&^* | 1 |  |  |  | 1 |  |  |  |  |  |  |  |  |  |  |  |  |
| 27 |  |  | *Gleditsia* | 1 |  | 1 |  |  |  |  |  |  |  | 1 |  |  |  |  |  |  |
| 28 |  |  | *Fagus* | 1 |  | 1 |  |  |  |  |  |  |  |  |  |  |  | 1 |  |  |
| 29 |  |  | *Quercus* | 2 |  | 1 |  | 1 |  |  |  | 1 |  | 1 |  |  |  |  |  |  |
| 30 |  |  | *Robinia* | 1 |  |  |  | 1 |  |  |  | 1 |  |  |  |  |  |  |  |  |
| 31 |  | Moracea | *Morus* | 1 |  |  | 1 |  |  |  | 1 |  |  |  |  |  |  |  |  |  |
| 32 |  | Rosaceae | *Crataegus* | 2 | 2 |  |  |  | 2 |  |  |  |  |  |  |  |  |  |  |  |
| 33 |  |  | *Malus* | 3 |  | 1 |  | 2 |  | 1 |  | 2 |  |  |  |  |  | 1 |  |  |
| 34 |  |  | *Padus* | 1 |  | 1 |  |  |  | 1 |  |  |  |  |  |  |  |  |  |  |
| 35 |  |  | *Prunus* | 3 |  |  |  | 3 |  |  |  | 2 |  |  |  |  |  |  |  | 1 |
| 36 |  |  | *Pyrus* | 2 |  |  |  | 2 |  |  |  | 2 |  |  |  |  |  |  |  |  |
| 37 | Saxifragales | Cercidiphyllaceae | *Cercidiphyllum* | 1 |  | 1 |  |  |  | 1 |  |  |  | 1 |  |  |  |  |  |  |
| 38 |  | Meliaceae | *Melia* | 1 |  |  | 1 |  |  |  | 1 |  |  |  |  |  |  |  |  |  |
| 39 |  |  | *Toona* | 1 |  |  |  | 1 |  |  |  | 1 |  |  |  |  |  |  |  |  |
| 40 |  | Sapindaceae | *Koelreuteria* | 2 |  | 1 |  | 1 |  | 1 |  | 1 |  |  |  |  |  |  |  |  |
| 41 |  |  | *Xanthoceras* | 1 |  | 1 |  |  |  | 1 |  |  |  |  |  |  |  |  |  |  |
| Total | 10 | 21 | 41 | 209 | 101 | 70 | 11 | 27 | 78 | 63 | 5 | 17 | 16 | 9 | 7 | 4 | 17 | 4 | 1 | 4 |

#: *Acer* contained 3 species from Korea, & the plant were reported in the Korea. HS: plant species on which ALB has been reported to complete its life cycle (from oviposition to emergence of new beetles), and it is also highly sensitive or very good host plant were recorded [21, 23, 34]; MS: plant species on which ALB has been completed its life cycle, but it was not recorded highly sensitive plant; PS: plant species on which ALB has been completed part of its life cycle, including feeding and oviposition, but the exit hole was recorded; LS: feeding or oviposition were only recorded in the reference [26]. The family and order to which the trees species belong were checked in the websites (<https://encyclopedia.thefreedictionary.com/>)

**References**

21. Wang, Z.G.; Huang, D.Z.; Yan, J.J. The sequence choice of the main tree species to *Anoplophora glabripennis* in northern part of China. *Journal of Agricultural University of Hebei* **2009**, *32*, 62-68, doi:10.3969/j.issn.1000-1573.2009.06.013.

23. Gao, H.Z.; Yang, X.Y.; Wei, J.N.; Lang, X.R. An Investigation on the resisance of major forestation species to *Anoplophora glabripennis* and *A. nobilis*. *Journal of Northwest Forestry College* **1997**, *12*, 42-46.

26. Meng, P.S.; Hoover, K.; Keena, M.A. Asian Longhorned Beetle (Coleoptera: Cerambycidae), an introduced pest of *Maple* and other hardwood trees in North America and Europe. *J. Integ. Pest Manag.* **2015**, *6*, 84-88, doi:10.1093/jipm/pmv003.

34. Hu, J.; Angeli, S.; Schuetz, S.; Luo, Y.; Hajek, A.E. Ecology and management of exotic and endemic Asian longhorned beetle *Anoplophora glabripennis*. *Agr. Forest Entomol.* **2009**, *11*, 359-375, doi:10.1111/j.1461-9563.2009.00443.x.
