# Supplementary material for: A Review of the Host Plant Location and Recognition Mechanisms of Asian Longhorn Beetle
Source: Insects. 2023 Mar 17;14(3):292. doi: 10.3390/insects14030292 (PMC10054519; doi:10.3390/insects14030292)
Supplement: Supplementary file 1 [file insects-14-00292-s001.zip › Supplementary files S3-Table S3.docx]

Table S3 Binding affinities of four recombinant *Agla*OBPs with different candidate ligands assessed by fluorescent competitive binding assays

| Ligands | *Agla*OPB1* |  | *Agla*OPB12 |  | *Agla*OPB45 |  | *Agla*OPB46 |  |
| --- | --- | --- | --- | --- | --- | --- | --- | --- |
|  | IC_50_ (μM/L) | Ki (μM/L) | IC_50_ (μM/L) | Ki (μM/L) | IC_50_ (μM/L) | Ki (μM/L) | IC_50_ (μM/L) | Ki (μM/L) |
| **1-NPN** |  | 7.65 |  | 3.25 |  | 3.37 |  | 3.25 |
| **Alcohols** | **12** |  |  |  |  |  |  |  |
| *Cis*-3-Hexen-1-ol | 6. 65 | **5. 88** | - | - | - | - | 8.82±0.35 | **6.34** |
| *Cis*-2-Hexen-1-ol | 9. 42 | 8. 33 |  |  |  |  |  |  |
| 1-butanol |  |  |  |  | 4.36±0.20 | **3.96** | - | - |
| 1-Hexanol | 13.90 | 12.29 | - | - | - | - |  |  |
| 1-Pentanol |  |  |  |  |  |  | 8.71±0.25 | **5.36** |
| 2-Pentanol |  |  |  |  | 1.15±0.13 | **0.96** | 2.37±0.20 | **2.12** |
| Benzyl alcohol |  |  | 21.84±3.06 | 16.70 | 1.05±0.011 | **0.92** | 1.03±0.12 | **0.82** |
| 1-Dodecanol |  |  | 2.56±0.15 | **1.96** | 18.31±0.26 | 13.31 | 1.00±0.11 | **0.74** |
| 1-tetradecanol |  |  | 1.25±0.20 | **0.96** | - | - | 18.12±2.21 | 15.01 |
| Farnesol |  |  | 1.35±0.18 | **1.03** | 3.87±0.18 | **2.81** | - | - |
| Linalool | 16. 78 | 14. 84 | - | - | - | - | - | - |
| Linalool oxide |  |  |  |  | 16.12±3.01 | 11.20 | - | - |
| **Aldehydes** | **8** |  |  |  |  |  |  |  |
| (*E*)-2-Hexenal | 14. 19 | 12.55 | - | - |  |  |  |  |
| Hexanal | 16. 22 | 14.35 | 9.30±0.38 | 7.11 |  |  |  |  |
| Heptanal | 14. 96 | 13.23 | 11.60±0.63 | 8.87 | 13.61±0.45 | 10.42 | 4.44±0.28 | **3.08** |
| Nonanal |  |  | - | - | 10.61±0.51 | 8.17 | 3.87±0.15 | **2.16** |
| Decanal | 20. 09 | 17.77 | 25.54±2.35 | 19.53 |  |  |  |  |
| (*E*)-2-Decenal | 13. 45 | 11. 90 |  |  |  |  |  |  |
| Dodecanal | 20. 32 | 17. 97 | 1.07±0.28 | **0.82** |  |  |  |  |
| Hexadecanal |  |  |  |  | 1.07±0.22 | **0.87** | 11.03±0.17 | 7.69 |
| **Esters** | **6** |  |  |  |  |  |  |  |
| Butyl caproate |  |  |  |  | 7.64±0.51 | **5.14** | - | - |
| *Cis*-3-Hexenyl acetate | 19.95 | 17.64 | 1.00±0.17 | **0.77** |  |  |  |  |
| Octyl acetate |  |  | 9.62±0.45 | 6.75 | - | - | - | - |
| Butyl hexanoate |  |  | 5.87±0.16 | 4.12 |  |  |  |  |
| Methyl jasmonate |  |  | 7.21±0.54 | 5.06 | - | - | - | - |
| **Terpenoids** | **6** |  |  |  |  |  |  |  |
| D-limonene | 19.35 | 17.11 |  |  | 13.15±0.41 | 10.01 | 3.57±0.19 | **2.01** |
| （*+*）-longifolene |  |  |  |  | 10.12±1.16 | 7.45 | 3.25±0.17 | **1.78** |
| *α*-Pinene | 10. 26 | 9.07 | - | - | 2.41±0.20 | **1.96** |  |  |
| *β*-Caryophyllene | 8.45 | **7.47** | 1.06±0.19 | **0.74** | - | - | - | - |
| *α*-Ocimene | 14.19 | 12.55 | 10.85±0.56 | 7.61 | 1.26±0.14 | **1.02** | - | - |
| （*-*）-camphene |  |  | - | - | 3.21±0.62 | **2.77** | 6.83±0.34 | **4.63** |

Notes: * the paper did not show the standard error in the reference, the other data is mean ± *SE*, 1-NPN: N-phenyl-1-naphthylamine, IC_50_: the concentration of ligand when half of the florescent 1-NPN was displaced, - indicated the IC_50_ value was not calculate. The data from: [78-79].

**References**

77. Li, G.; Chen, X.; Shang, T. cDNA cloning, expression and ligand binding properties of the odorant binding protein *Agla*OBP12 in the Asian longhorned beetle, *Anoplopha glabripennis* (Coleoptera: Cerambycidae). *Acta Entomologica Sinica* **2017**, *60*, 1141-1154, doi:10.16380/j.kcxb.2017.10.005.

78. Li, G.; Chen, X.; Shang, T.; Yao, F. Cloning, expression, and binding properties of odorant binding protein 1 from *Anoplophora glabripennis* Motschulsky (Coleoptera: Cerambycidae). *Journal of Environmental Entomology* **2017**, *39*, 919-929, doi:10.3969/j.issn.1674-0858.2017.04.25.

79. Wang, Q. Identification of olfactory related genes and functional analysis of the key odorant binding proteins from two species of *Anoplophora*. Doctor thesis, Beijing Forestry University, Beijing, 2019.
