# Supplementary material for: A Review of the Host Plant Location and Recognition Mechanisms of Asian Longhorn Beetle
Source: Insects. 2023 Mar 17;14(3):292. doi: 10.3390/insects14030292 (PMC10054519; doi:10.3390/insects14030292)
Supplement: Supplementary file 1 [file insects-14-00292-s001.zip › Supplementary files S4-Table S4.docx]

Table S4 The diversity in sex pheromone composition of the ALB

| Function types | Chemical composite | Test insects | Sources | Gland | References |
| --- | --- | --- | --- | --- | --- |
| Volatile pheromone | 4-(n-heptyloxy)butanal, 4-(n-heptyloxy)butanol | Female | Male | - | [95] |
| Volatile or aggregation pheromone | (3*E*, 6*E*)-α-farnesene | Adult | Male | - | [81] |
| Contact sex pheromone | (*Z*)-9-tricosene, (*Z*)-7-pentacosene, (*Z*)-9-pentacosene, (*Z*)-7-heptacosene, (*Z*)-9-heptacosene | male | Female | - | [96] |
| Long-range sex pheromone | Heptanal, nonanal, hexadecanal, octadecanal, eicosanal | Male | Female | - | [98] |
| Trail pheromones | Major: 2-methyldocosane; (*Z*)-9-tricosene | Male | Female | - | [104] |
|  | Minor: (*Z*)-9-pentacosene; (*Z*)-7-pentacosene |  |  |  |  |
| Volatile pheromone | Major: *α*-longipinene  Minor: *α*-cubebene, *α*-ylangene, (−)-*α*-copaene, *α*-bergamotene, *β*-caryophyllene, and *α*-farnesene | Adult | Female | Genitalia | [80] |

Notes: - indicate the glands that produce the substance are not reported in references, but Hank and Millar (2016) suggest that aggregation sex pheromones from male are produced by glands in the prothorax [105].

**References**

80. Xu, T.; Hansen, L.; Cha, D.H.; Hao, D.; Zhang, L.; Teale, S.A. Identification of a female-produced pheromone in a destructive invasive species: Asian longhorn beetle, *Anoplophora glabripennis*. *J. Pest Sci.* **2020**, *93*, 1321-1332, doi:10.1007/s10340-020-01229-3.

81. Crook, D.J.; Lance, D.R.; Mastro, V.C. Identification of a potential third component of the male-produced pheromone of *Anoplophora glabripennis* and its effect on behavior. *J. Chem. Ecol.* **2014**, *40*, 1241-1250, doi:10.1007/s10886-014-0520-3.

95. Zhang, A.; Oliver, J.E.; Aldrich, J.R.; Wang, B.; Mastro, V.C. Stimulatory beetle volatiles for the Asian longhorned beetle, *Anoplophora glabripennis* (Motschulsky). *Zeitschrift fur Naturforschung C* **2002**, *57*, 553-558, doi:10.1515/znc-2002-5-626.

96. Zhang, A.; Oliver, J.E.; Chauhan, K.; Zhao, B.; Xia, L.; Xu, Z. Evidence for contact sex recognition pheromone of the Asian longhorned beetle, *Anoplophora glabripennis* (Coleoptera: Cerambycidae). *Naturwissenschaften* **2003**, *90*, 410-413, doi:10.1007/s00114-003-0452-1.

98. Wickham, J.D.; Xu, Z.; Teale, S.A. Evidence for a female-produced, long range pheromone of *Anoplophora glabripennis* (Coleoptera: Cerambycidae). *Insect Sci.* **2012**, *19*, 355-371, doi:10.1111/j.1744-7917.2012.01504.x.

104. Hoover, K.; Keena, M.; Nehme, M.; Wang, S.; Meng, P.; Zhang, A. Sex-specific trail pheromone mediates complex mate finding behavior in *Anoplophora glabripennis*. *J. Chem. Ecol.* **2014**, *40*, 169-180, doi:10.1007/s10886-014-0385-5.

105. Hanks, L.M.; Millar, J.G. Sex and aggregation-sex pheromones of Cerambycid beetles: basic science and practical applications. *J. Chem. Ecol.* **2016**, *42*, 631-654, doi:10.1007/s10886-016-0733-8.
