# Supplementary material for: A Review of the Host Plant Location and Recognition Mechanisms of Asian Longhorn Beetle
Source: Insects. 2023 Mar 17;14(3):292. doi: 10.3390/insects14030292 (PMC10054519; doi:10.3390/insects14030292)
Supplement: Supplementary file 1 [file insects-14-00292-s001.zip › Supplementary files S5-PPT S1.pptx]

## Slide 1
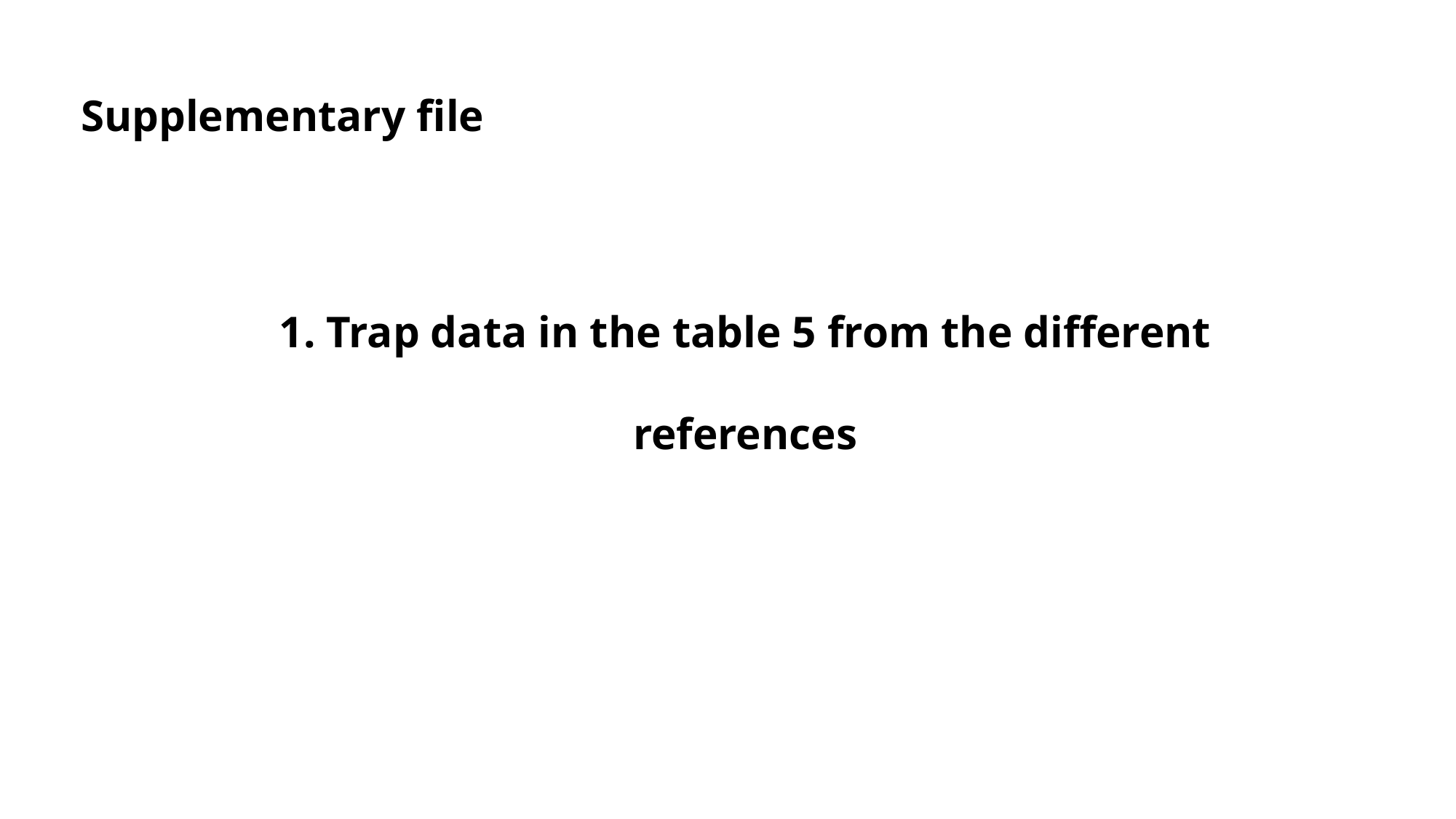

Supplementary file
1. Trap data in the table 5 from the different references

## Slide 2
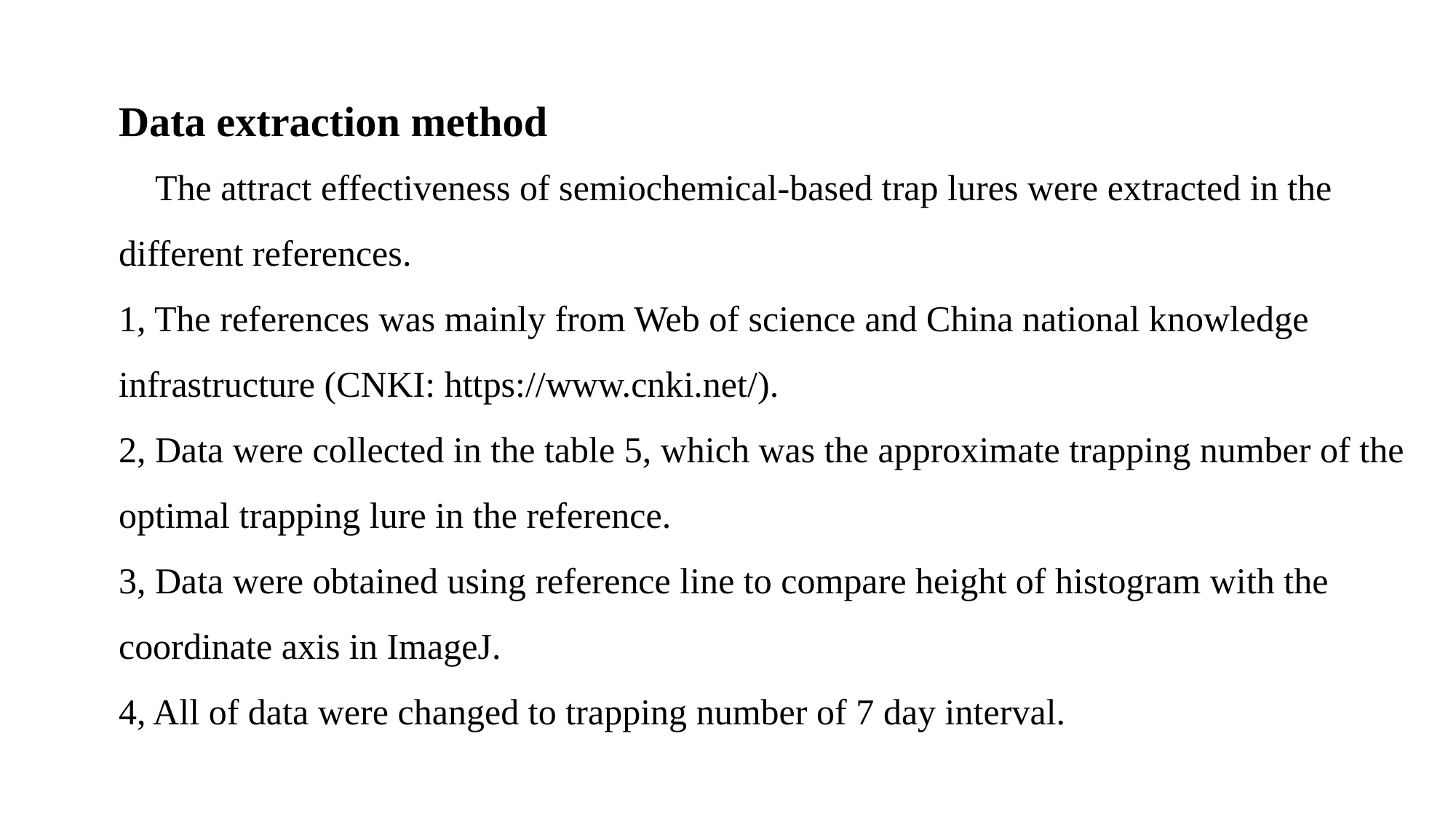

Data extraction method
 The attract effectiveness of semiochemical-based trap lures were extracted in the different references.
1, The references was mainly from Web of science and China national knowledge infrastructure (CNKI: https://www.cnki.net/).
2, Data were collected in the table 5, which was the approximate trapping number of the optimal trapping lure in the reference.
3, Data were obtained using reference line to compare height of histogram with the coordinate axis in ImageJ.
4, All of data were changed to trapping number of 7 day interval.

## Slide 3
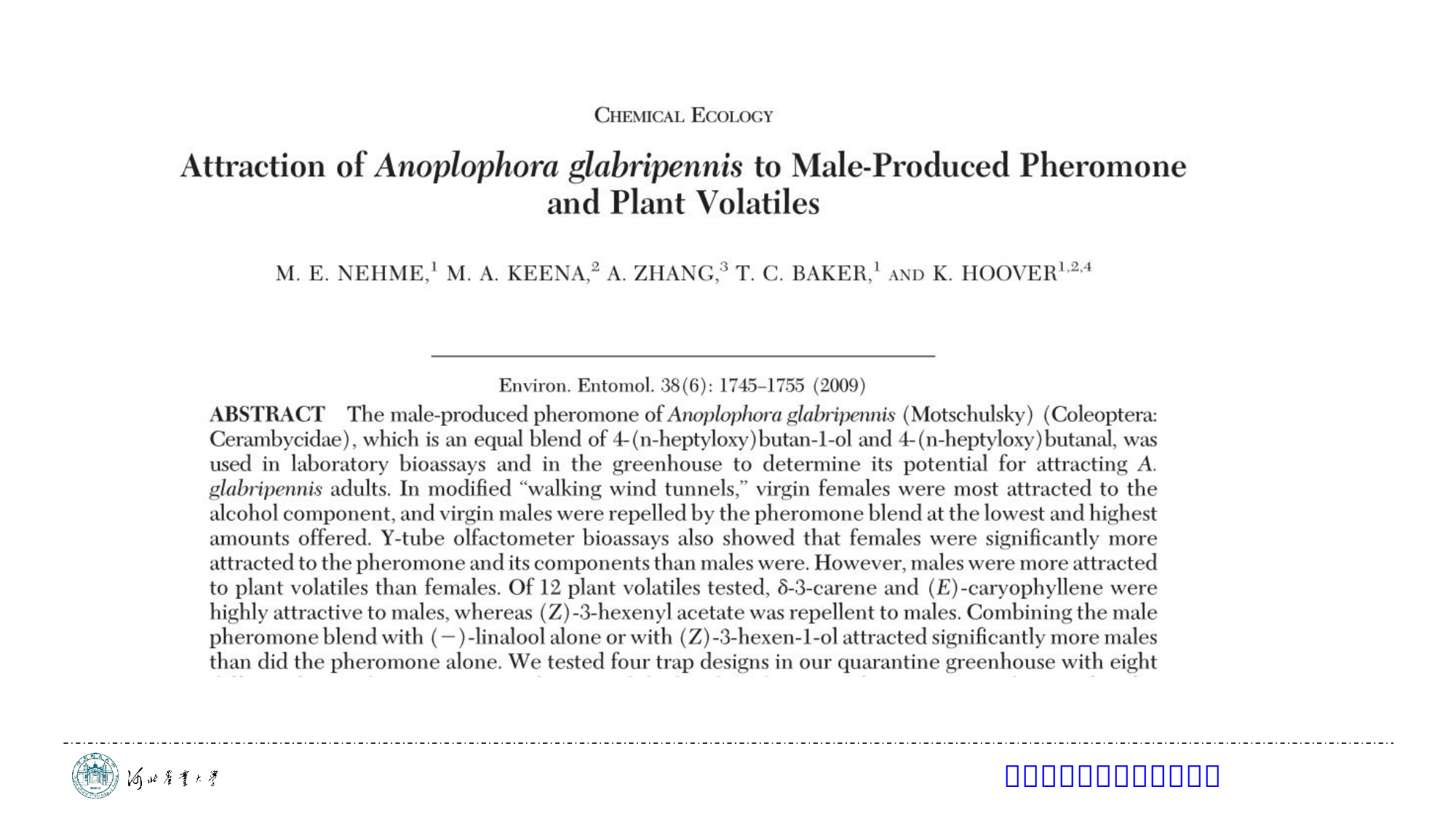

林木害虫无公害防控实验室

## Slide 4
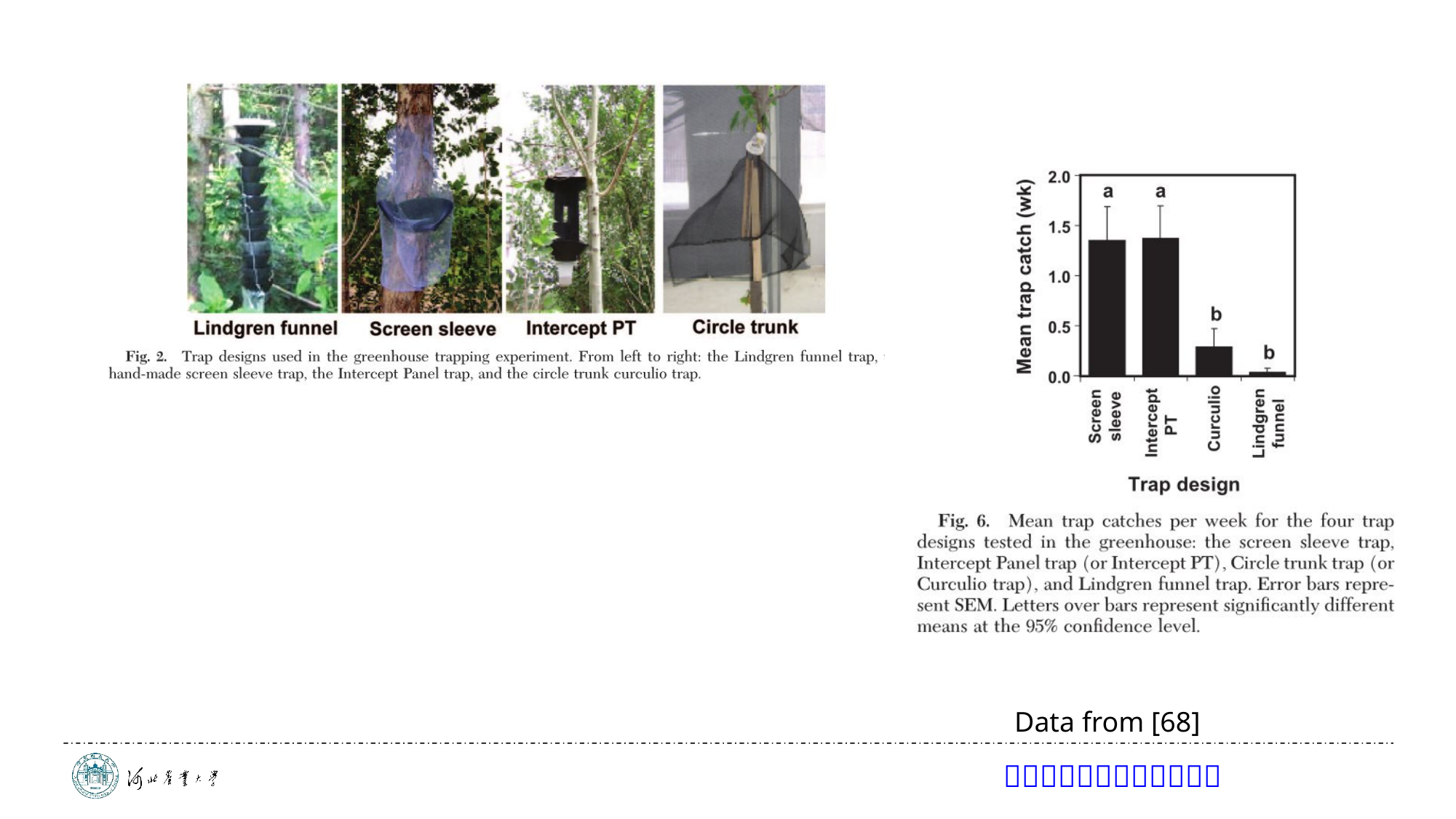

Data from [68]
林木害虫无公害防控实验室

## Slide 5
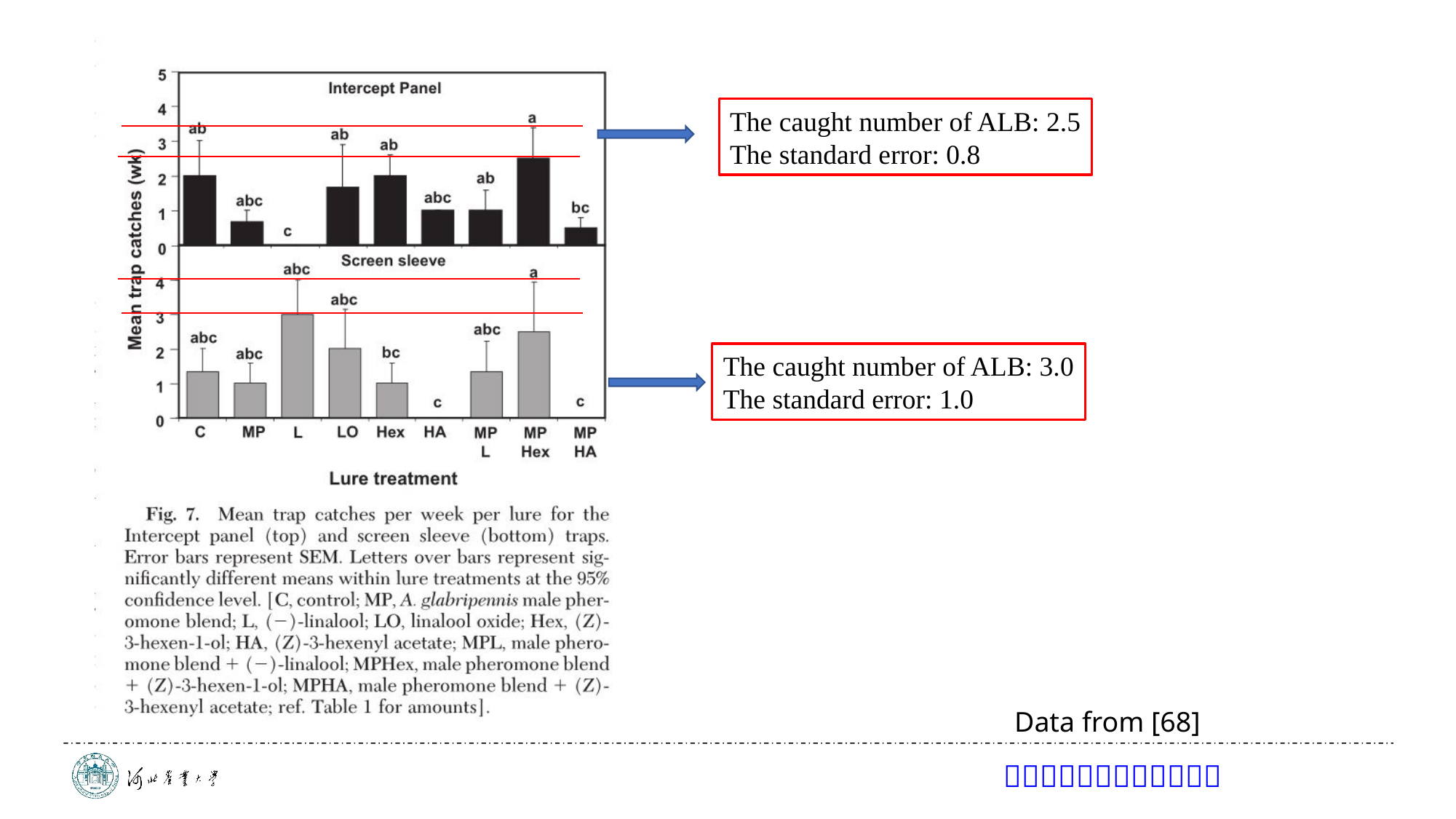

The caught number of ALB: 2.5
The standard error: 0.8
The caught number of ALB: 3.0
The standard error: 1.0
Data from [68]
林木害虫无公害防控实验室

## Slide 6
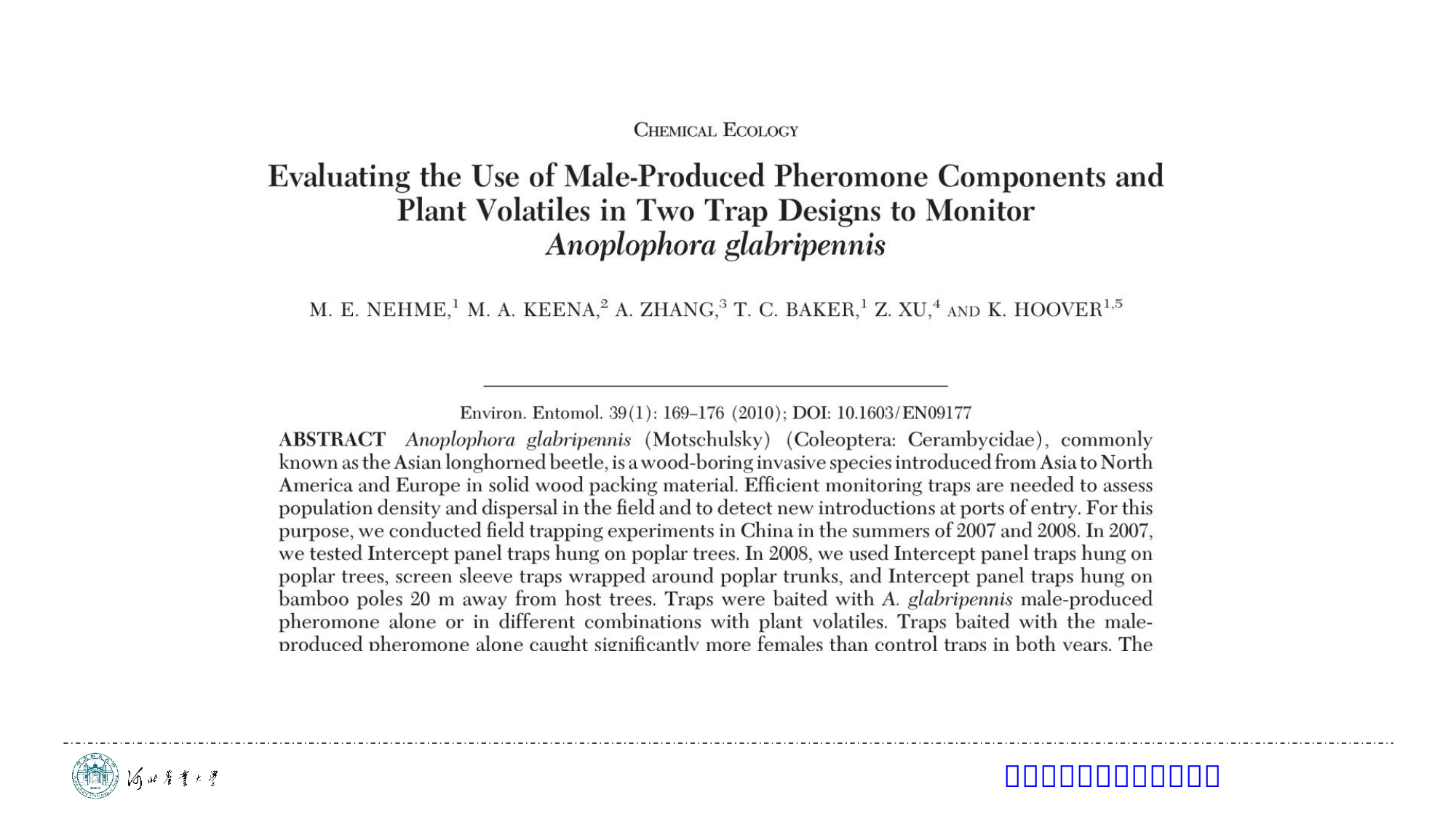

林木害虫无公害防控实验室

## Slide 7
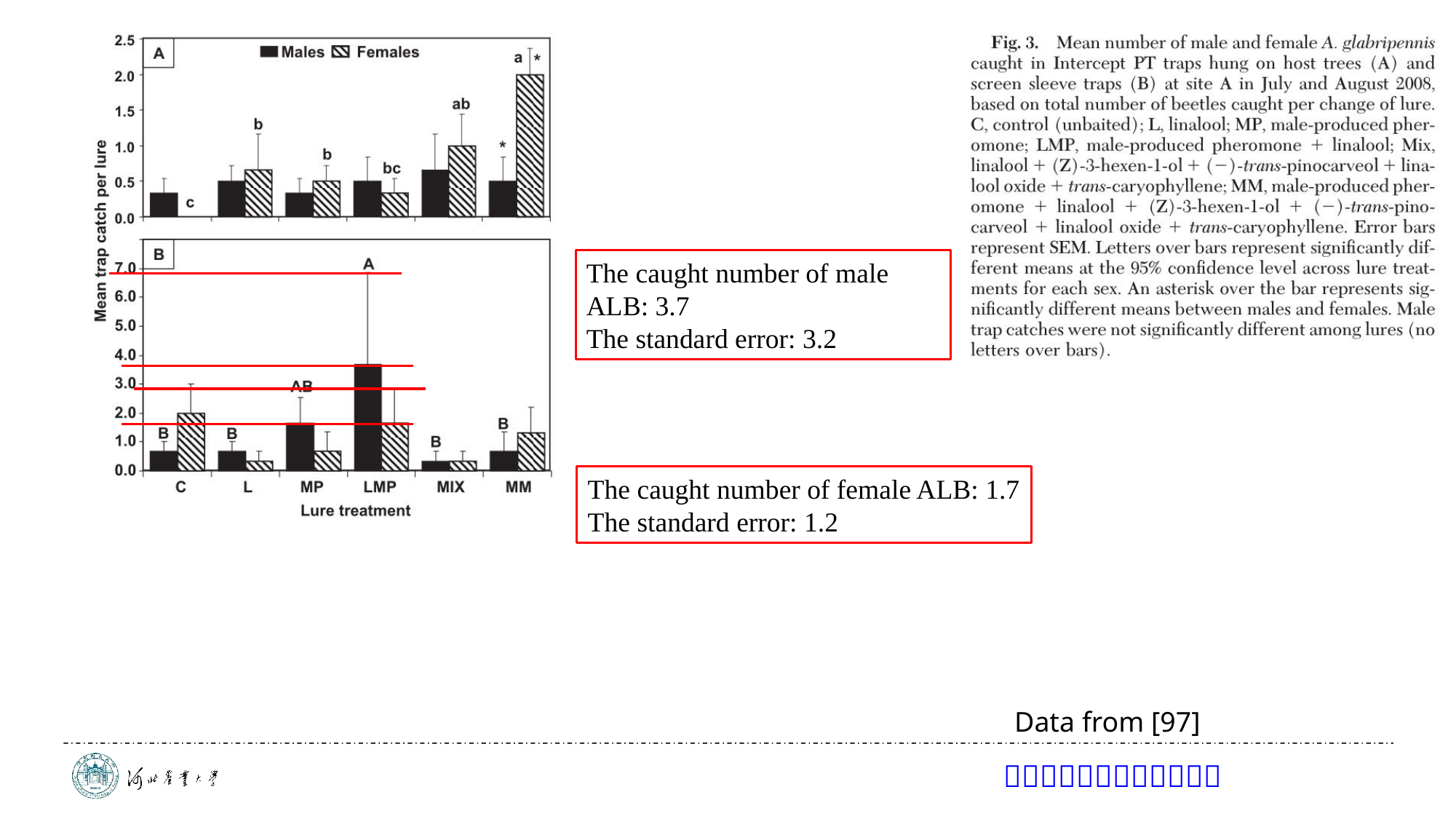

The caught number of male ALB: 3.7
The standard error: 3.2
The caught number of female ALB: 1.7
The standard error: 1.2
Data from [97]
林木害虫无公害防控实验室

## Slide 8
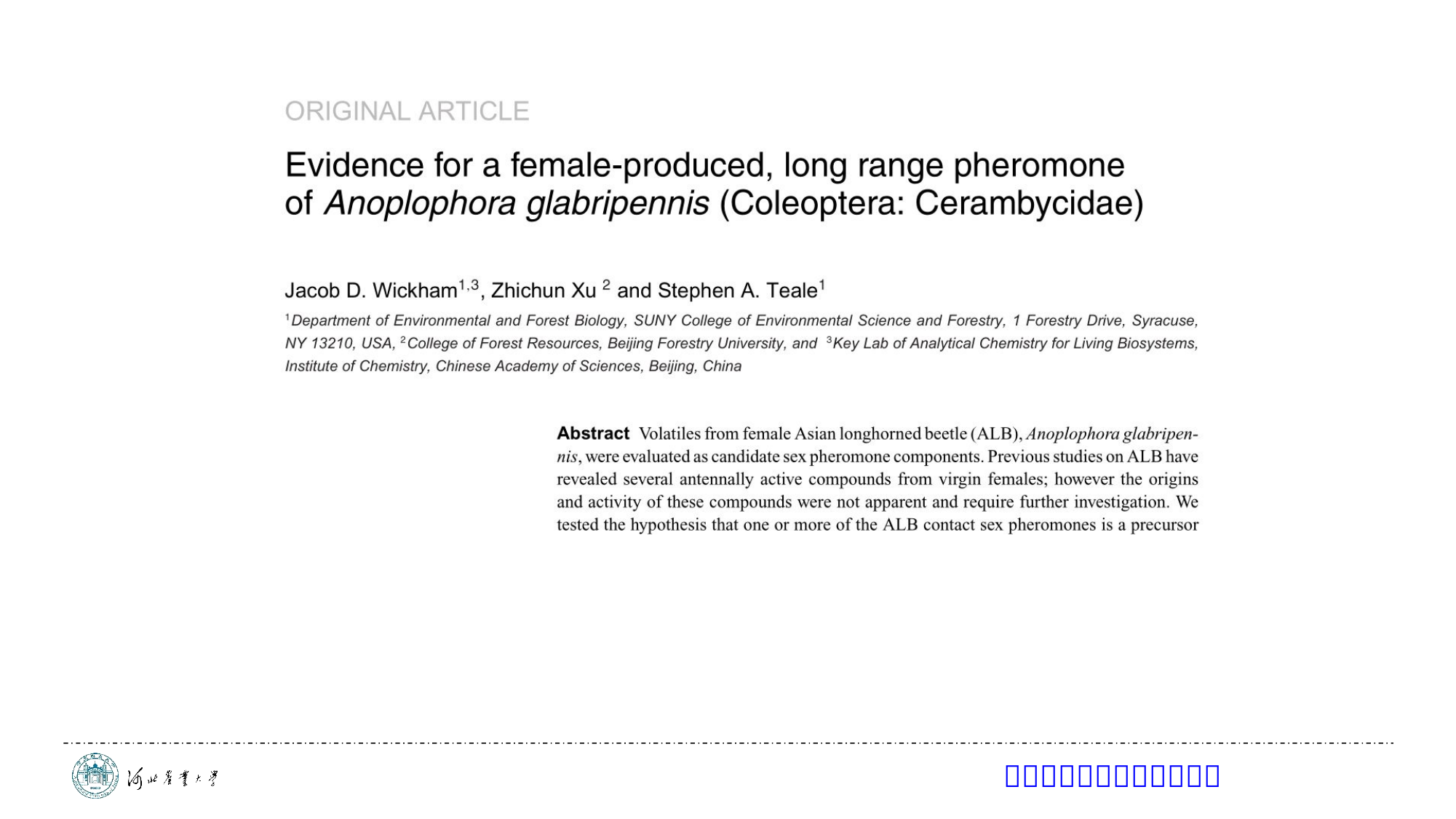

林木害虫无公害防控实验室

## Slide 9
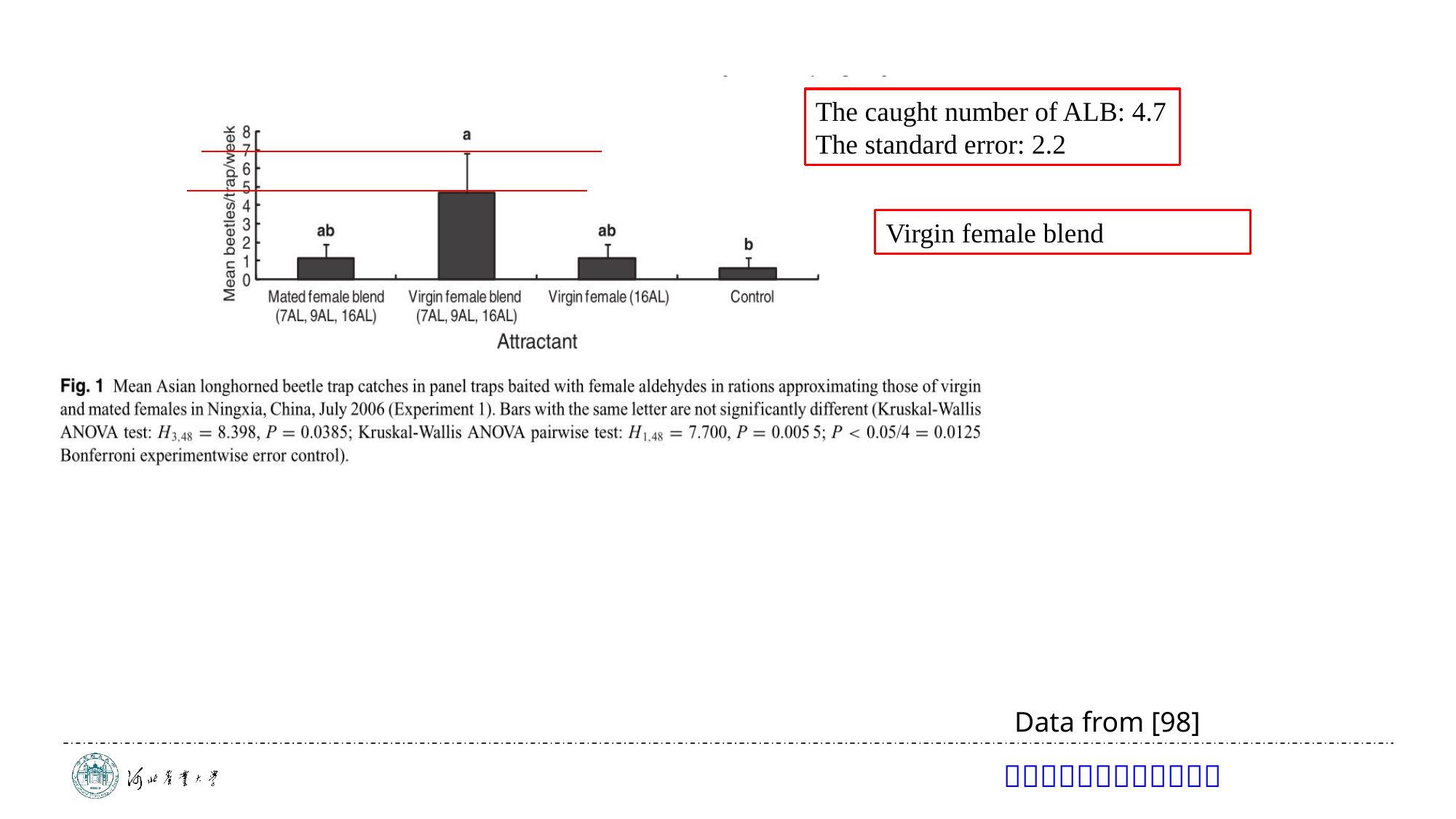

The caught number of ALB: 4.7
The standard error: 2.2
Virgin female blend
Data from [98]
林木害虫无公害防控实验室

## Slide 10
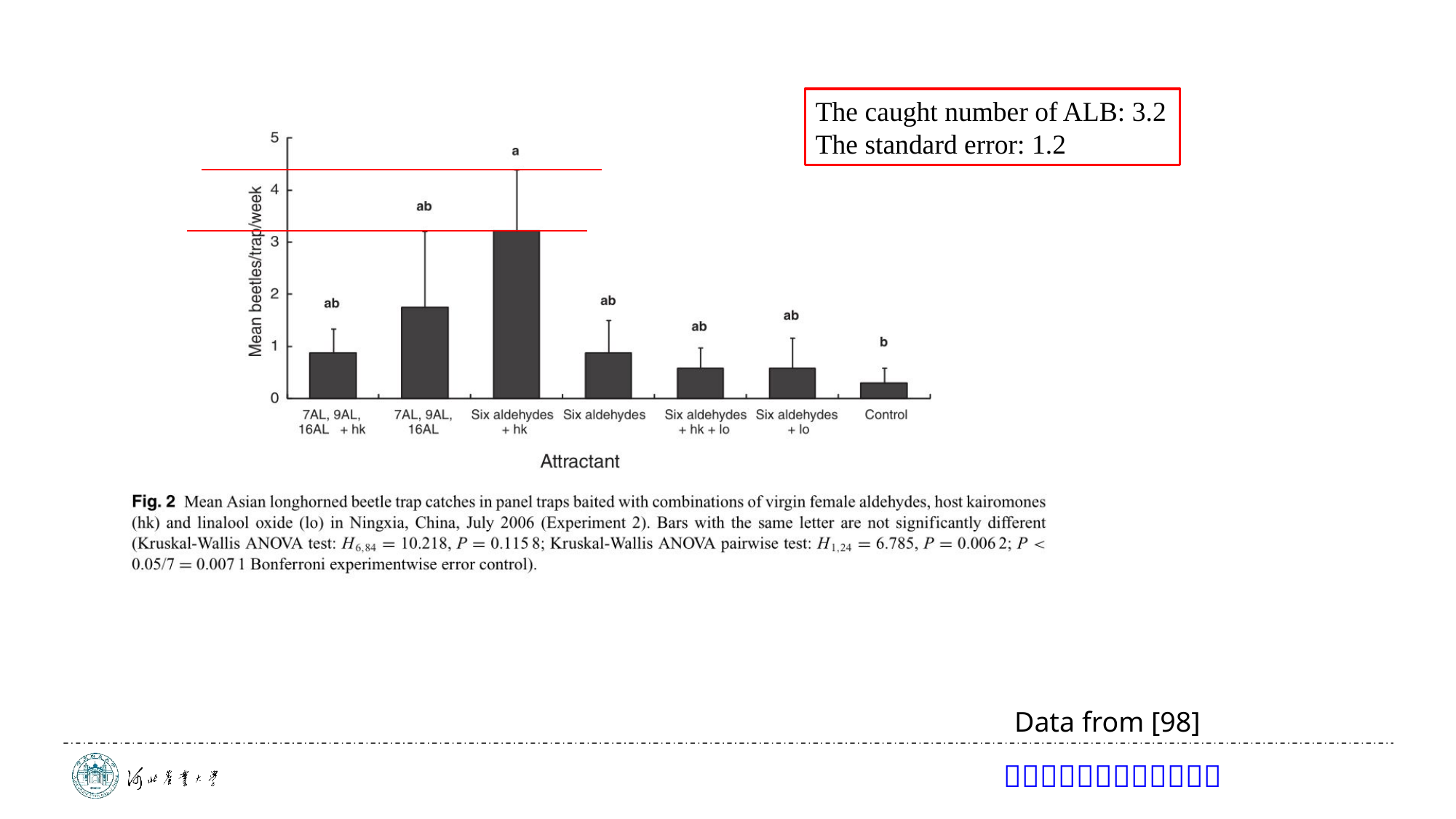

The caught number of ALB: 3.2
The standard error: 1.2
Data from [98]
林木害虫无公害防控实验室

## Slide 11
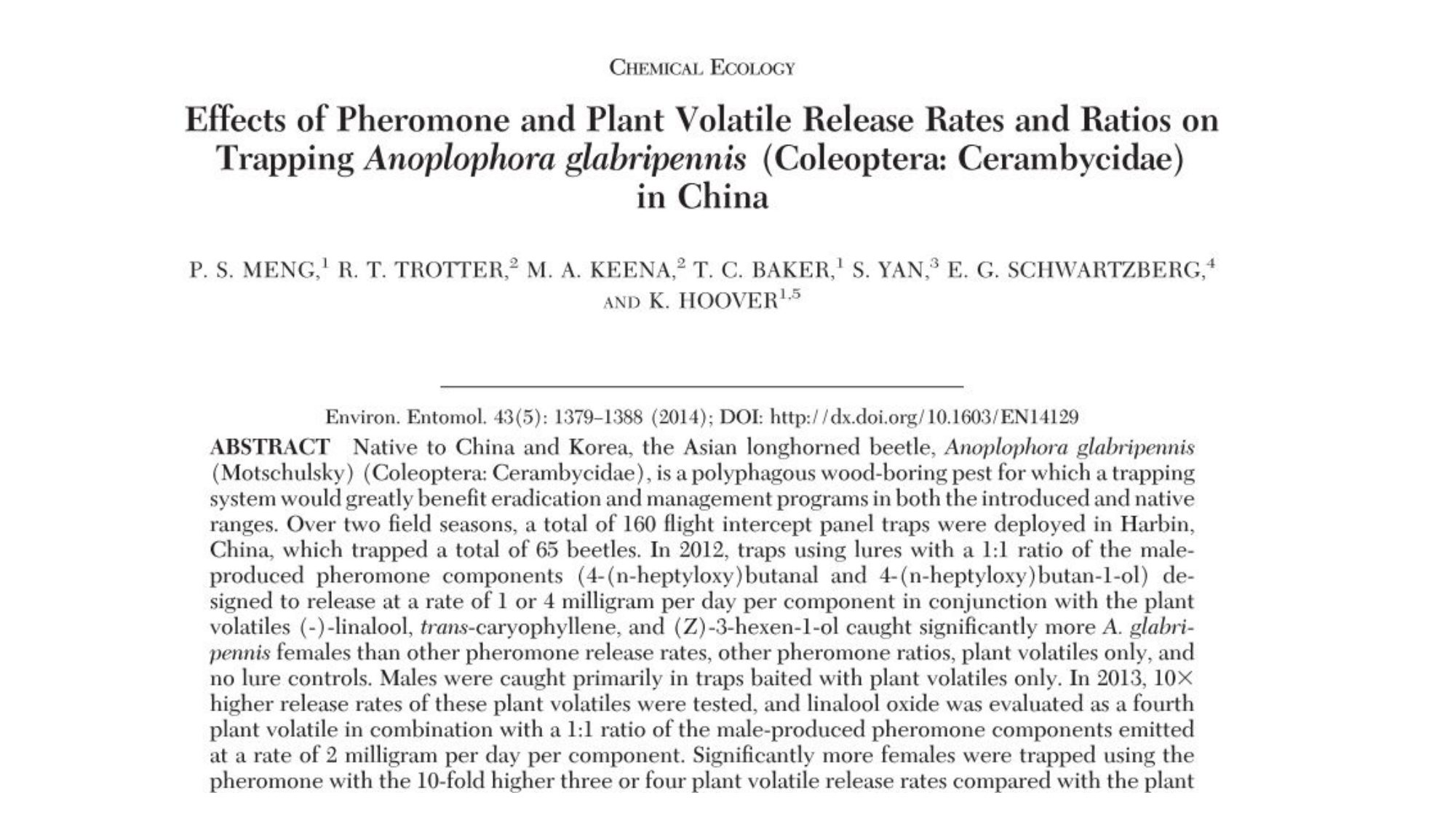

## Slide 12
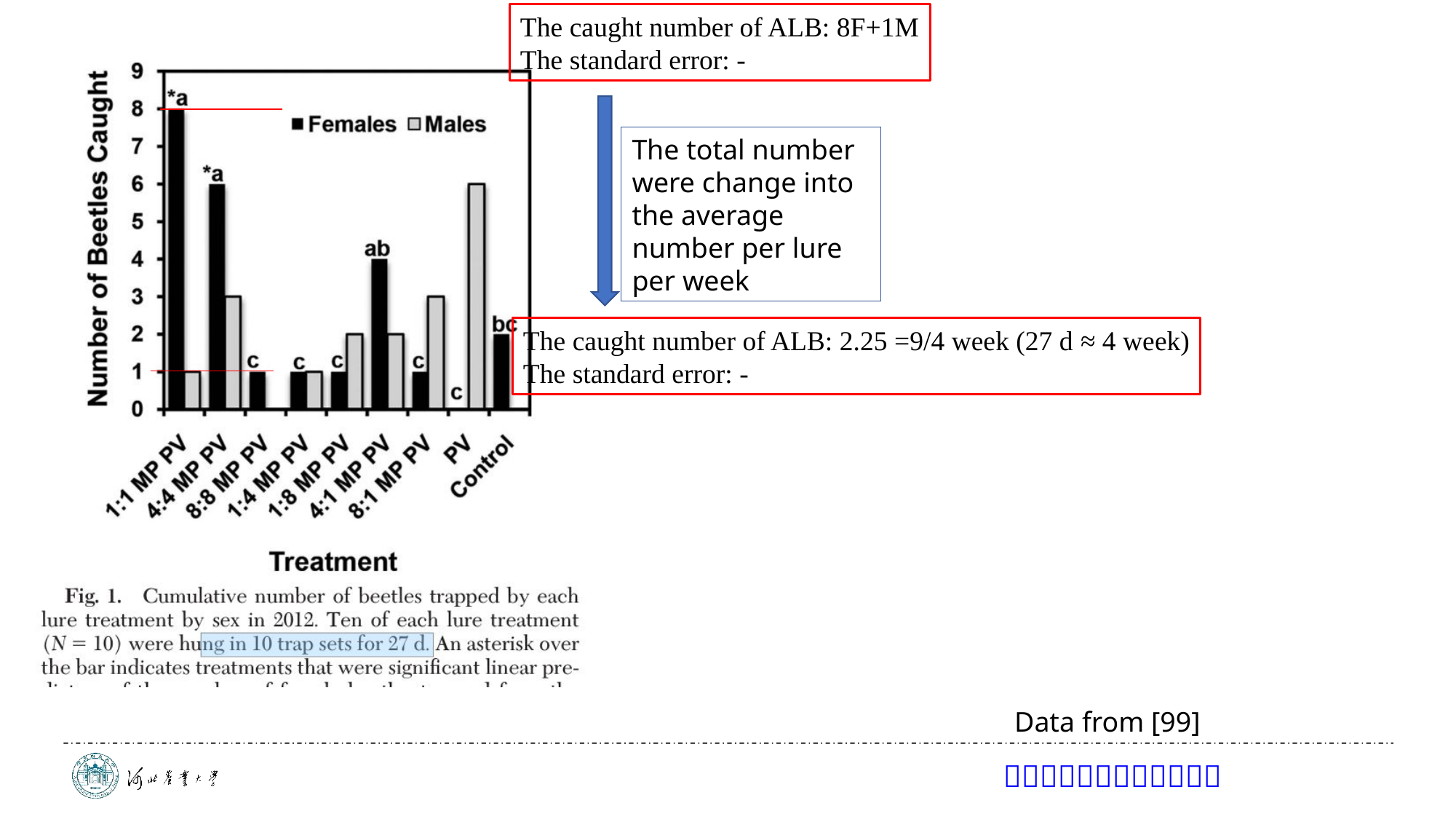

The caught number of ALB: 8F+1M
The standard error: -
The total number were change into the average number per lure per week
The caught number of ALB: 2.25 =9/4 week (27 d ≈ 4 week)
The standard error: -
Data from [99]
林木害虫无公害防控实验室

## Slide 13
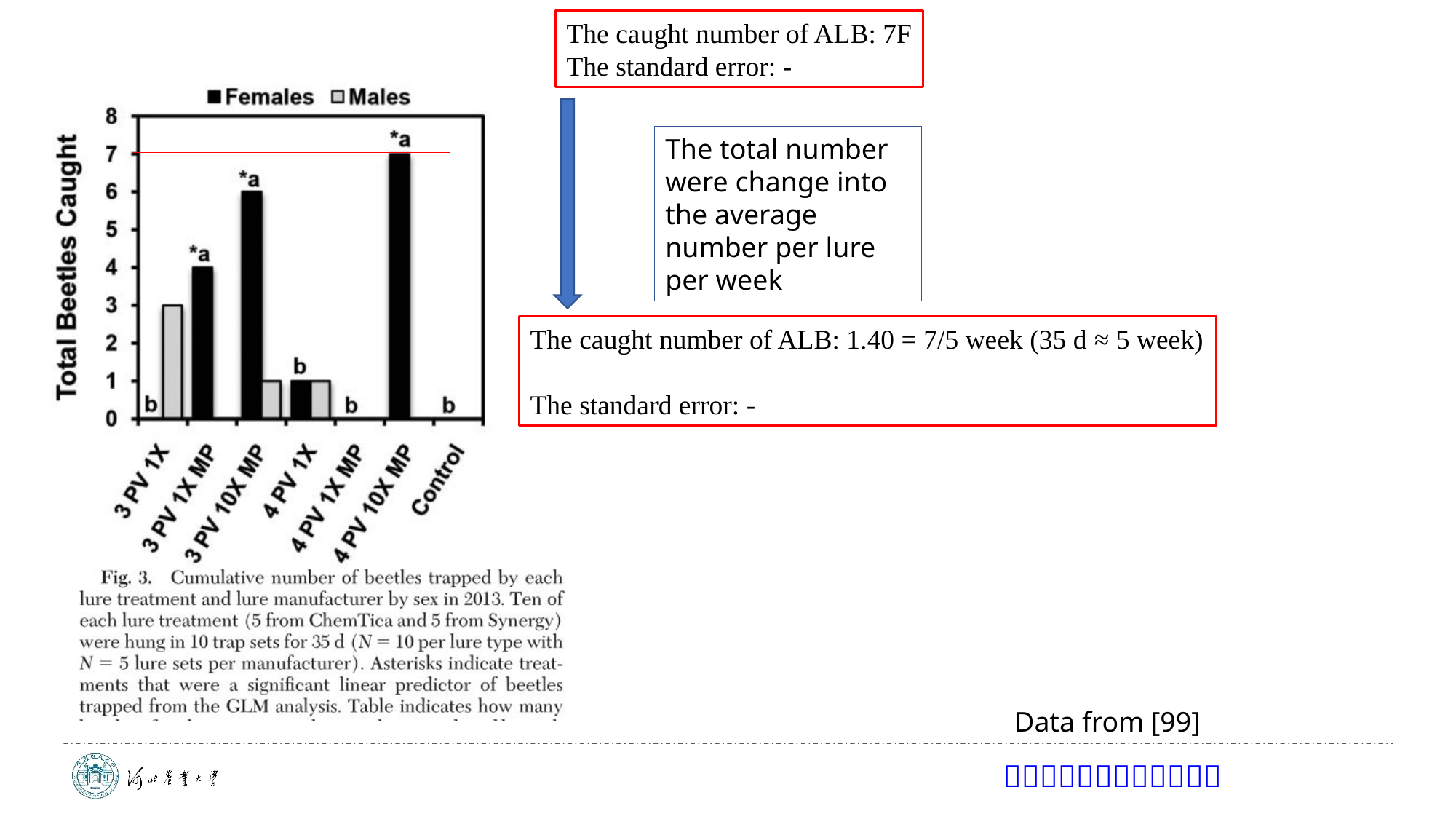

The caught number of ALB: 7F
The standard error: -
The total number were change into the average number per lure per week
The caught number of ALB: 1.40 = 7/5 week (35 d ≈ 5 week)
The standard error: -
Data from [99]
林木害虫无公害防控实验室

## Slide 14
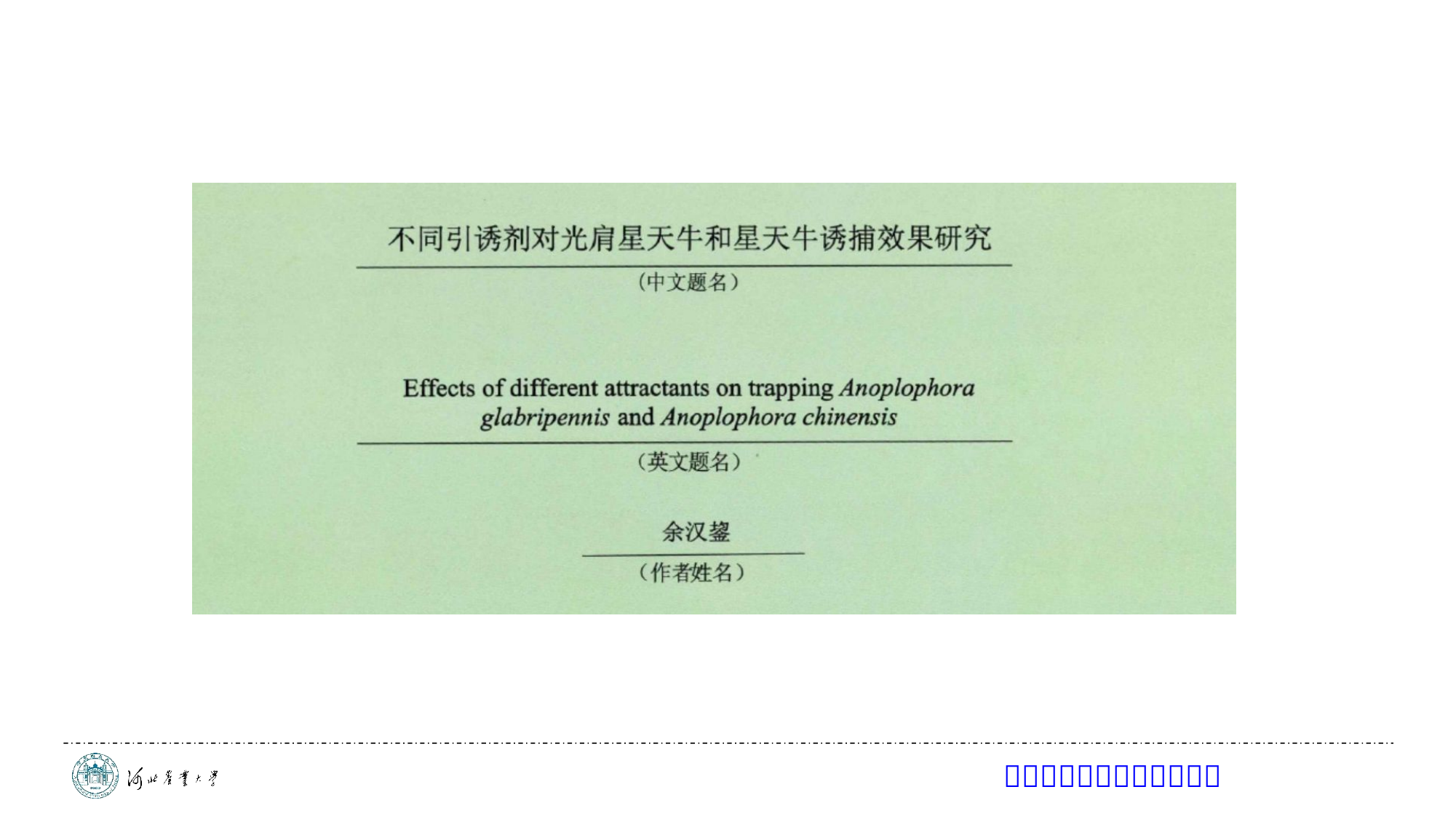

林木害虫无公害防控实验室

## Slide 15
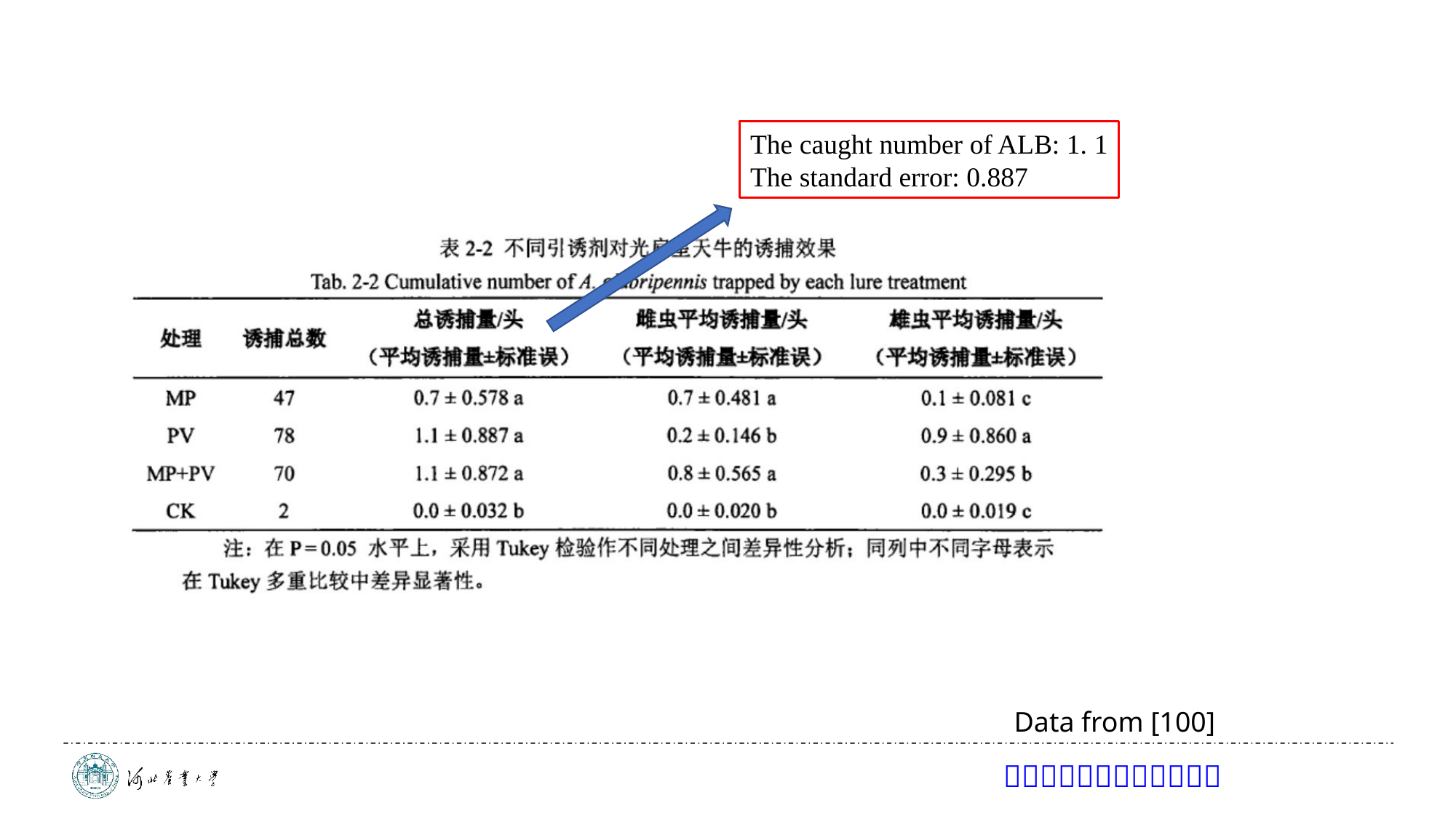

The caught number of ALB: 1. 1
The standard error: 0.887
Data from [100]
林木害虫无公害防控实验室

## Slide 16
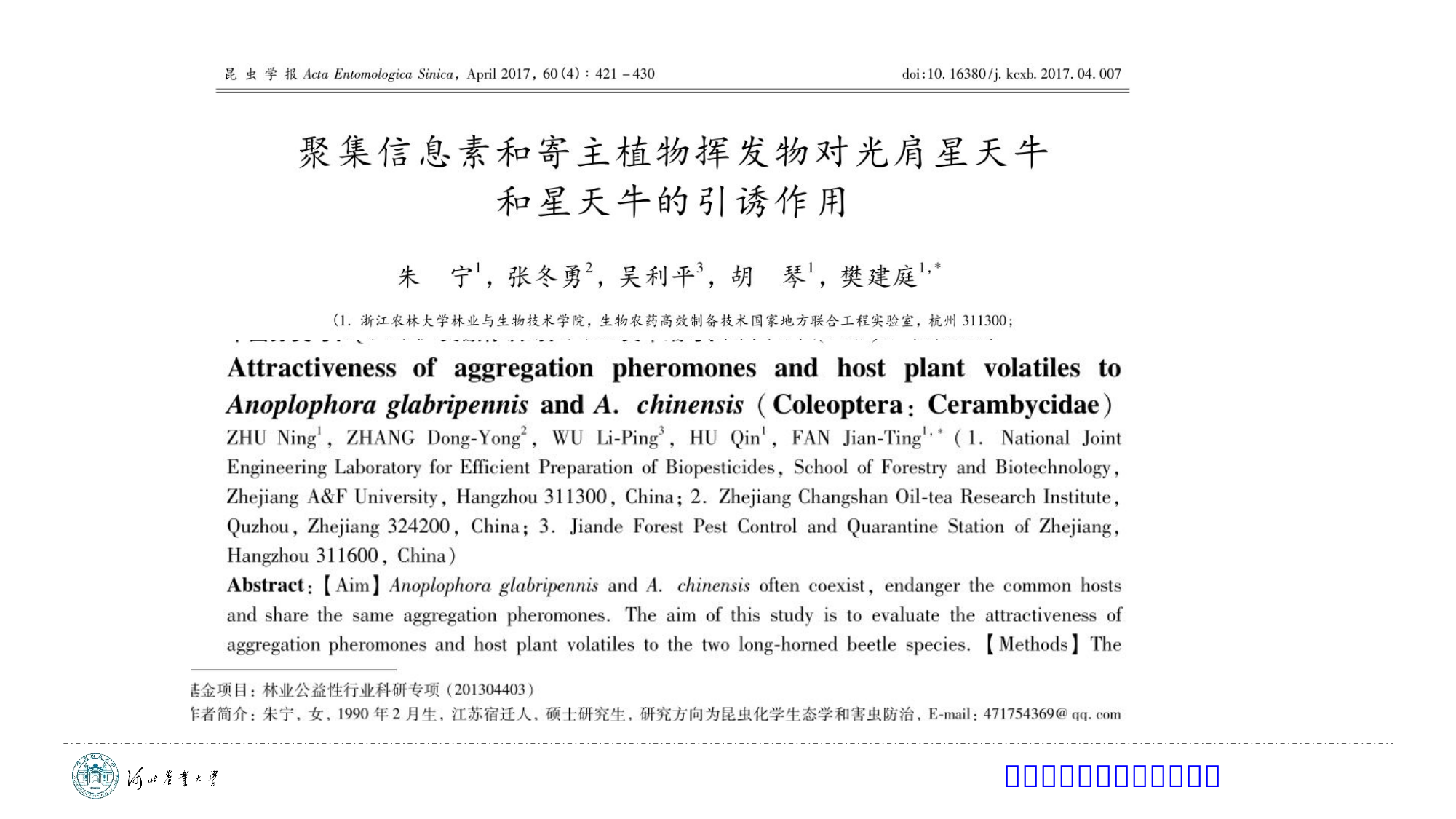

林木害虫无公害防控实验室

## Slide 17
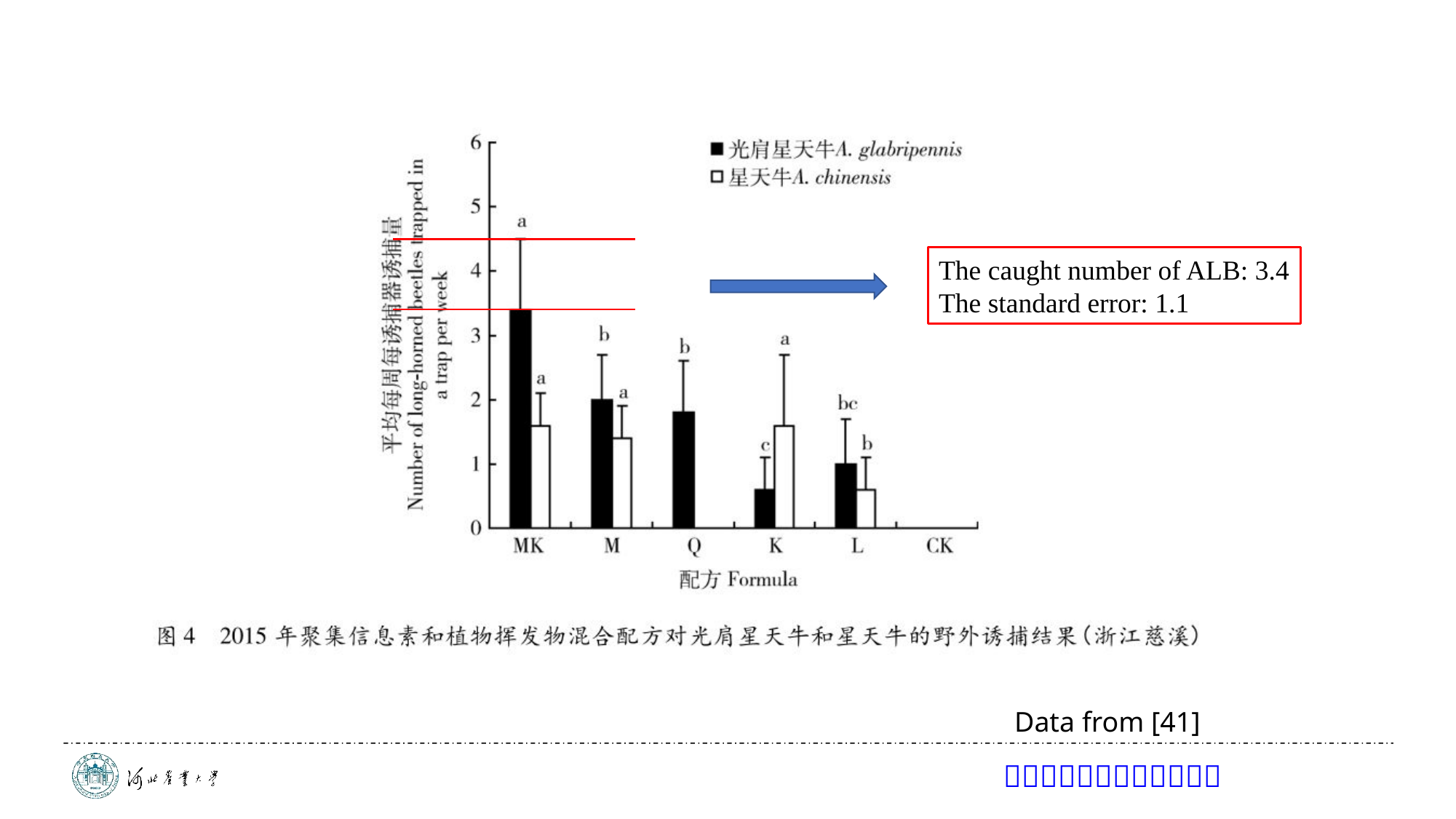

The caught number of ALB: 3.4
The standard error: 1.1
Data from [41]
林木害虫无公害防控实验室

## Slide 18
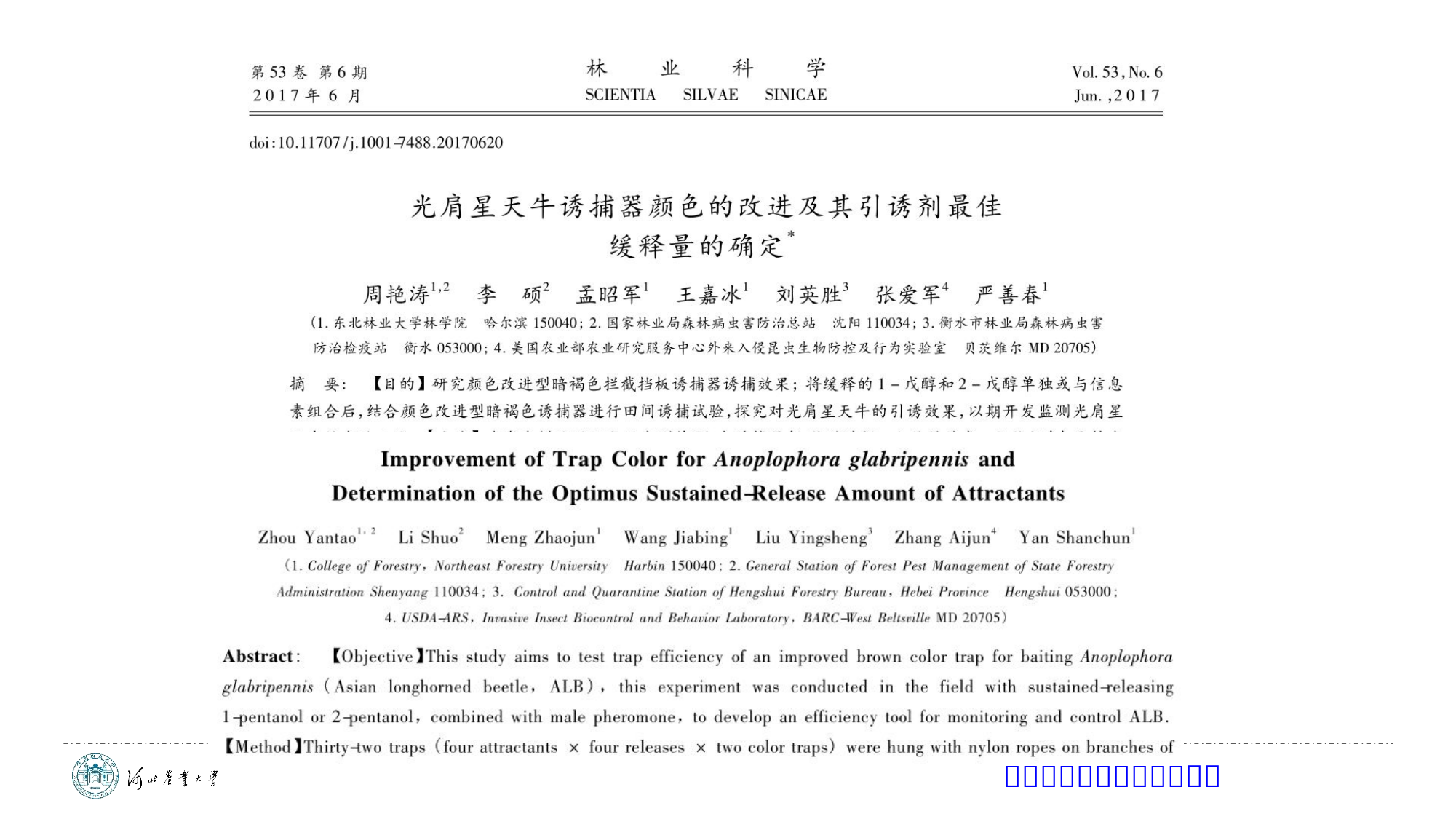

林木害虫无公害防控实验室

## Slide 19
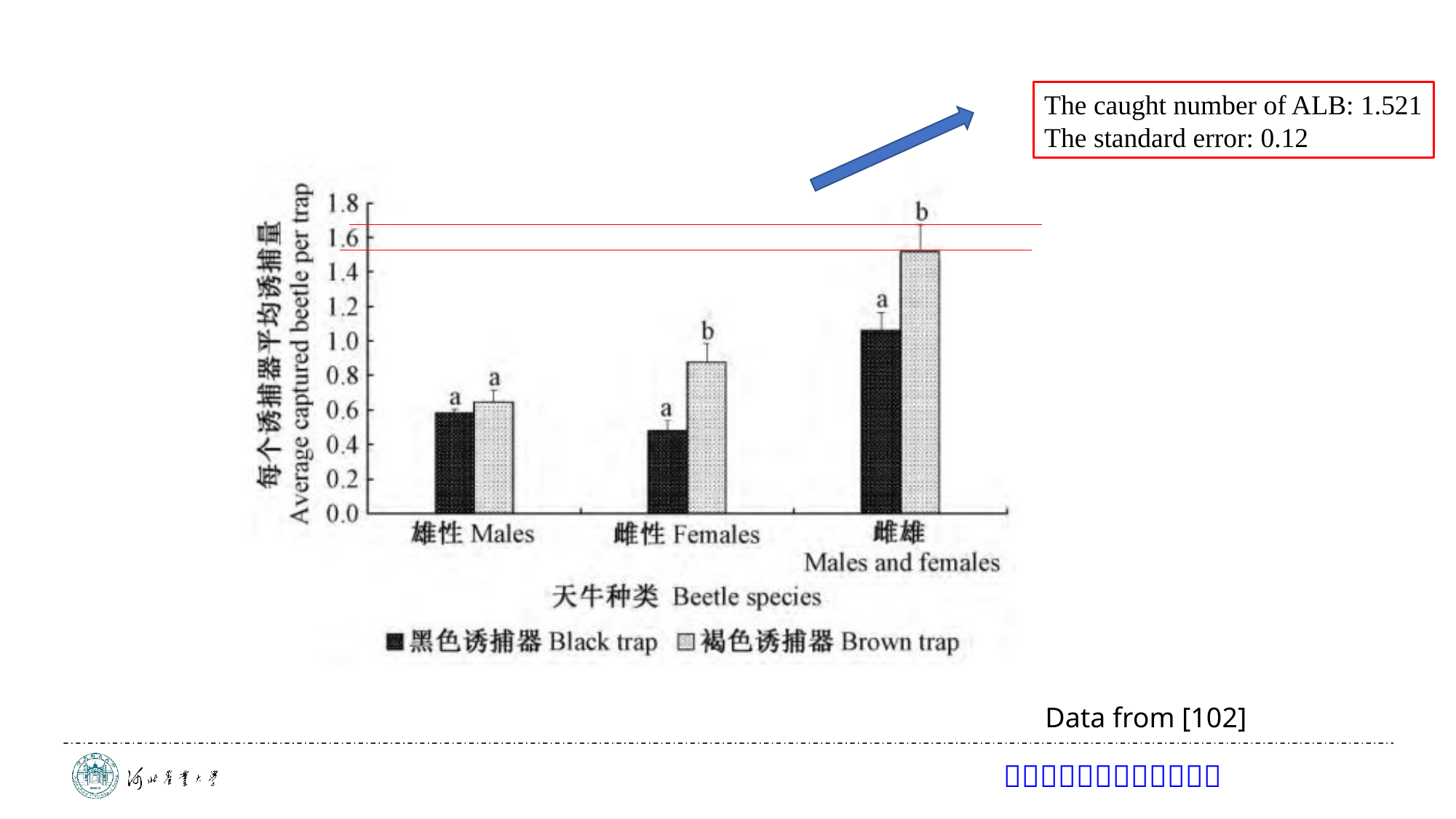

The caught number of ALB: 1.521
The standard error: 0.12
Data from [102]
林木害虫无公害防控实验室

## Slide 20
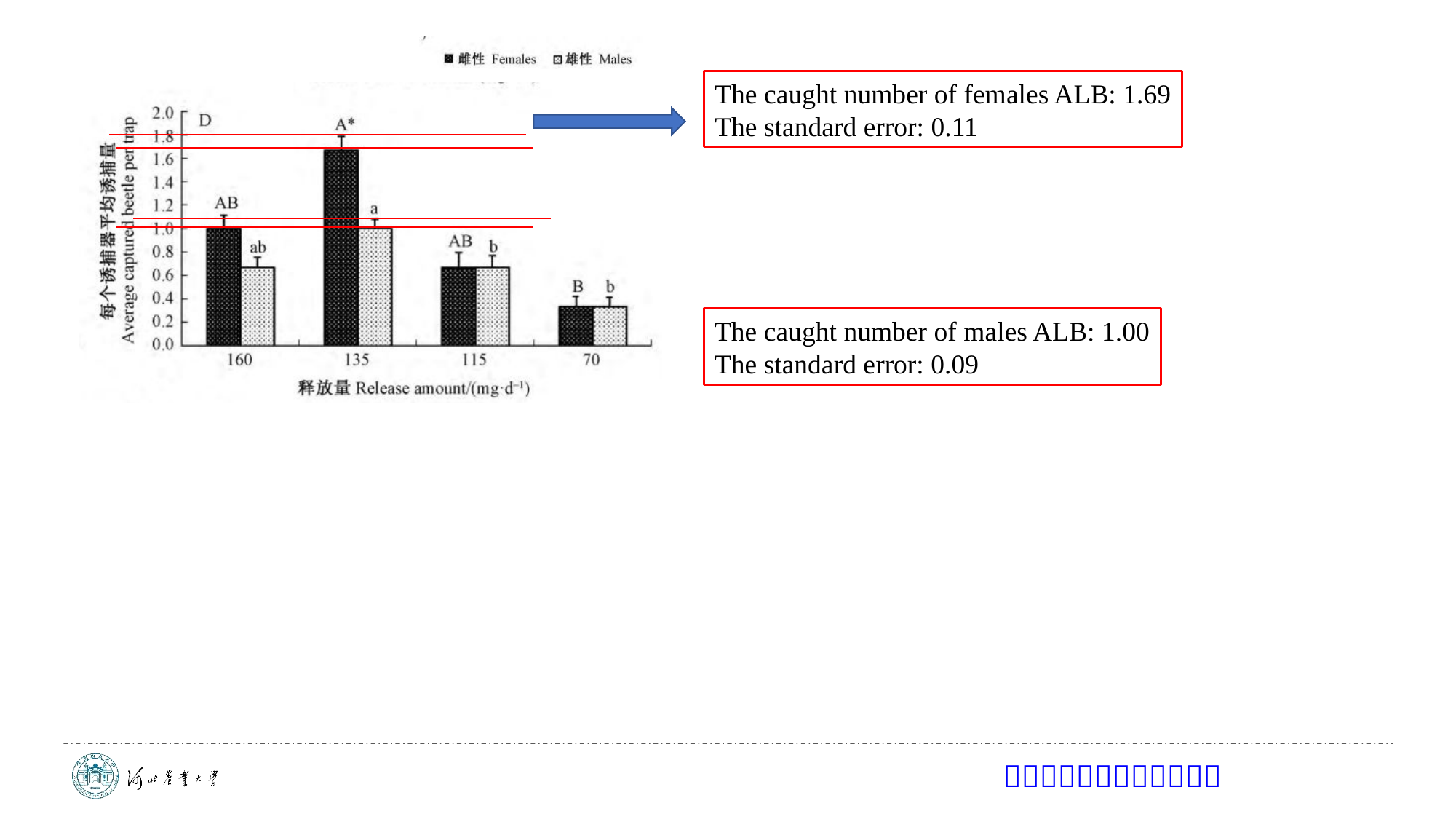

The caught number of females ALB: 1.69
The standard error: 0.11
The caught number of males ALB: 1.00
The standard error: 0.09
林木害虫无公害防控实验室

## Slide 21
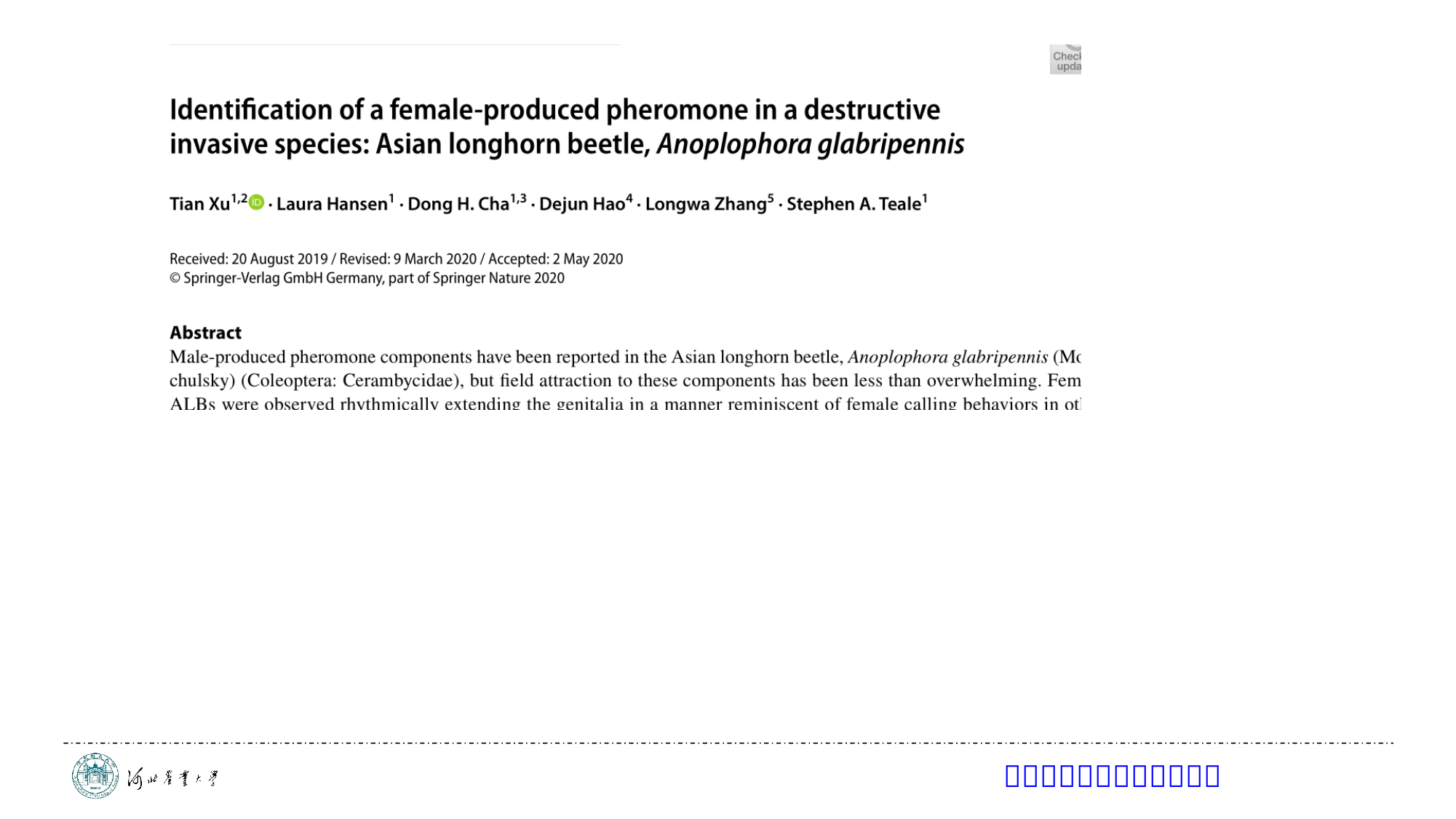

林木害虫无公害防控实验室

## Slide 22
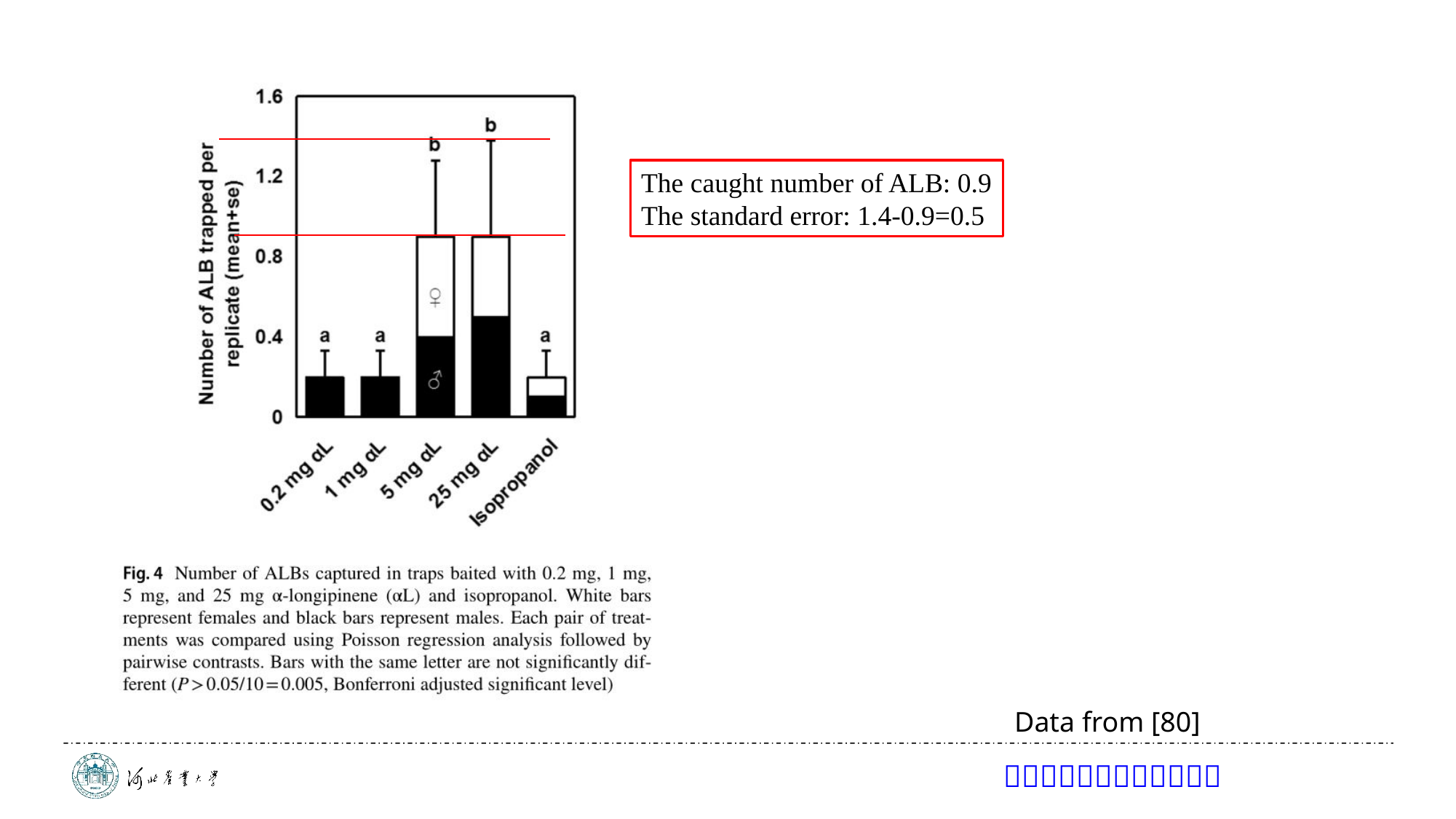

The caught number of ALB: 0.9
The standard error: 1.4-0.9=0.5
Data from [80]
林木害虫无公害防控实验室

## Slide 23
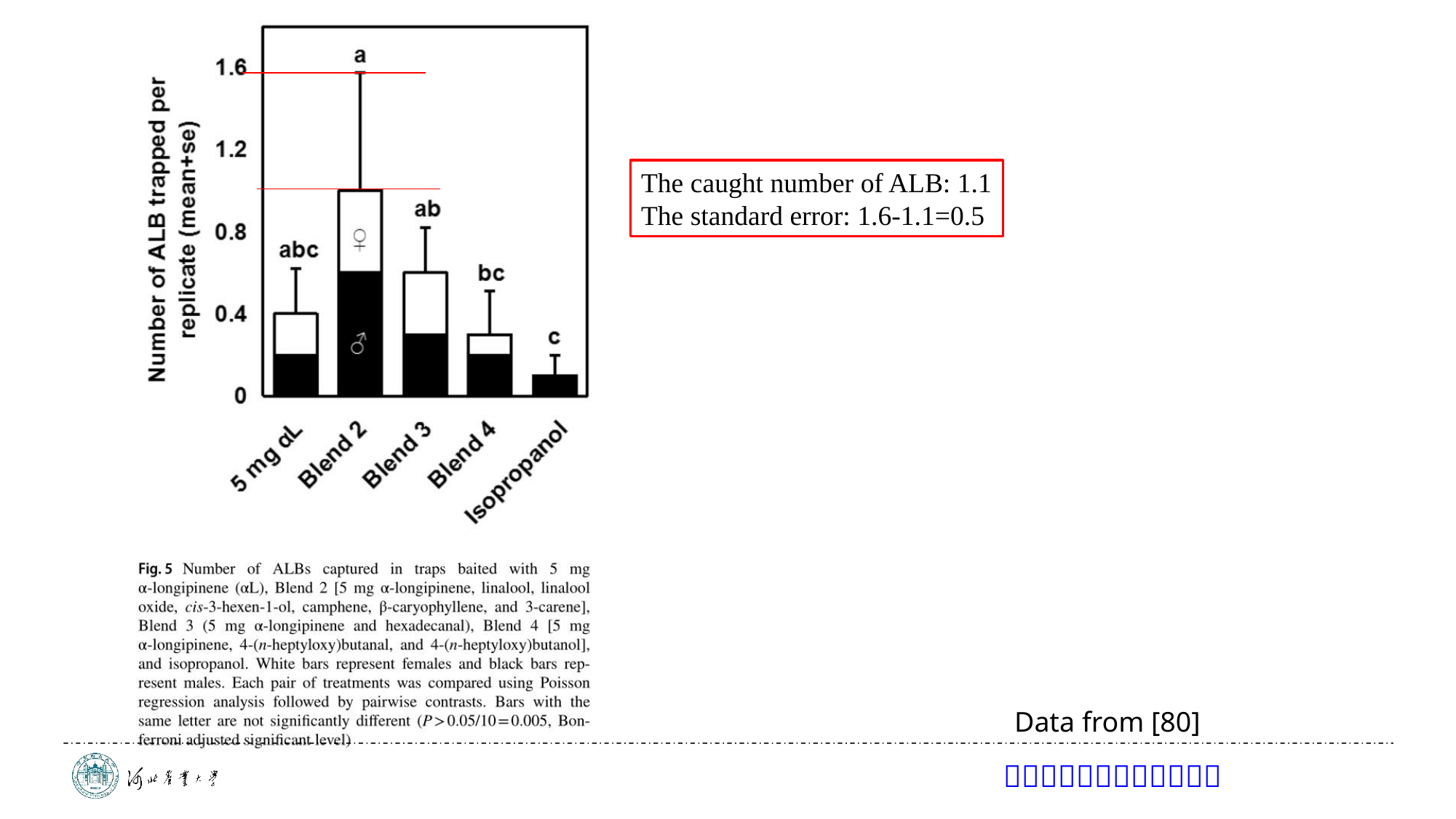

The caught number of ALB: 1.1
The standard error: 1.6-1.1=0.5
Data from [80]
林木害虫无公害防控实验室
